# Single‐cell profiling reveals tumour cell heterogeneity accompanying a pre‐malignant and immunosuppressive microenvironment in gastric adenocarcinoma

**DOI:** 10.1002/ctm2.1490

**Published:** 2023-11-30

**Authors:** Jie Ge, Xiao Xiao, Haiyan Zhou, Mimi Tang, Jing Bai, Xinchen Zou, Chunliu Zhang, Changhao Huang, Xiang Feng, Ting Liu, Xin Yi, Xuefeng Xia, Heli Liu, Zihua Chen

**Affiliations:** ^1^ Department of Gastrointestinal Surgery Xiangya Hospital Central South University Changsha China; ^2^ Genomics Institute Geneplus‐Shenzhen Shenzhen China; ^3^ Department of Pathology Xiangya Hospital Central South University Changsha China; ^4^ Institute for Rational and Safe Medication Practices National Clinical Research Center for Geriatric Disorders Xiangya Hospital Central South University Changsha China; ^5^ Geneplus‐Beijing Institute Beijing China; ^6^ The Hunan Provincial Key Laboratory of Precision Diagnosis and Treatment for Gastrointestinal Tumor Xiangya Hospital Central South University Changsha China; ^7^ International Joint Research Center of Minimally Invasive Endoscopic Technology Equipment & Standardization Xiangya Hospital, Central South University Changsha China

To the Editor:

Gastric adenocarcinoma (GAC) is a leading cause of cancer‐related disease and death.^1^ Current phenotypical classifications, however, lack precision in guiding therapy decisions.^2^ The challenge lies in the molecular and morphologic heterogeneity within and across tumours. Herein, we performed single‐cell RNA sequencing (scRNA‐seq) and single‐cell T‐cell receptor sequencing (scTCR‐seq) on nine treatment‐naive GAC patients, including different Lauren's subtypes (Figure 1A and Figure S1A; Table S1).

**FIGURE 1:**
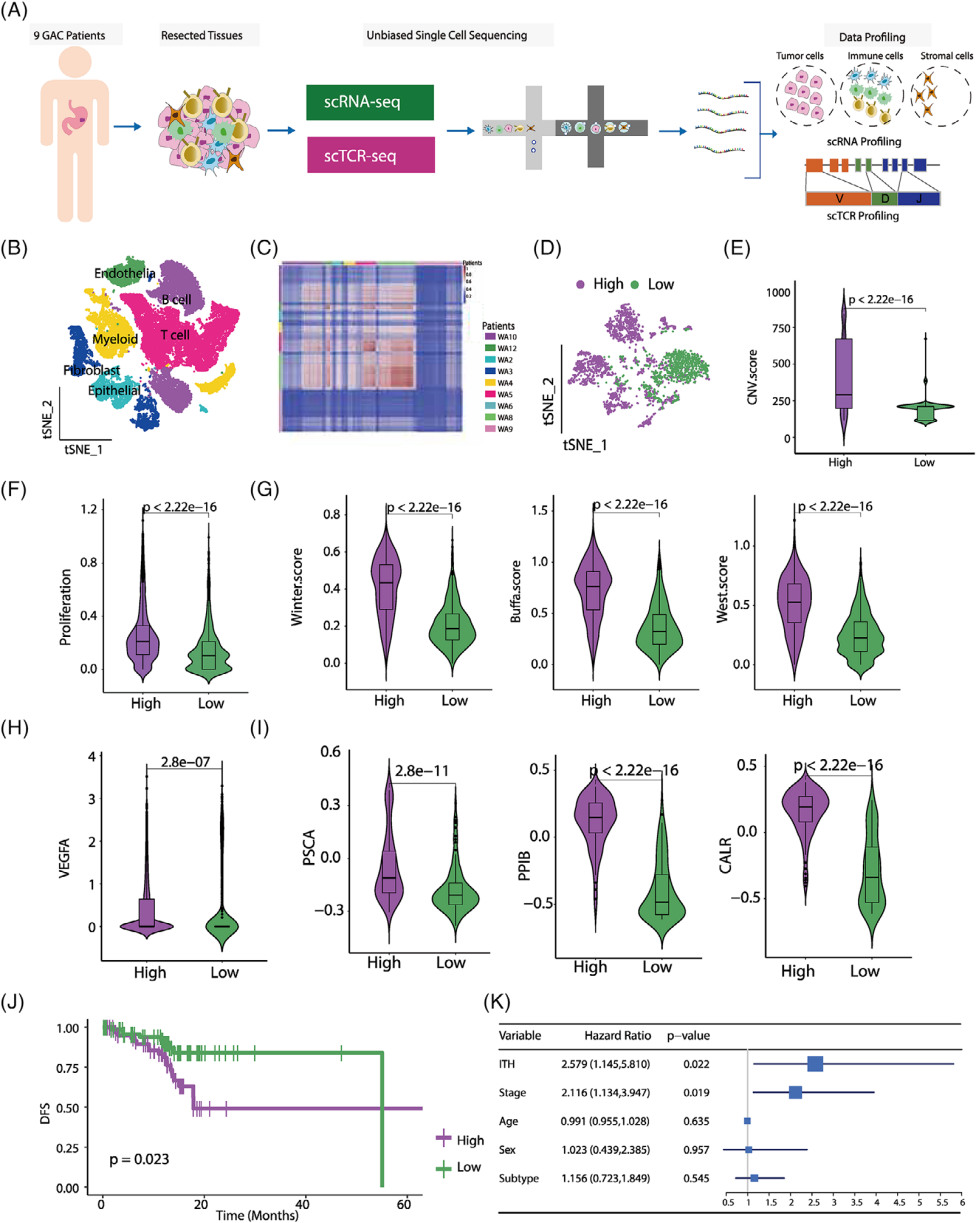
Association between intratumour heterogeneity (ITH) of cancer cells and patient outcome. (A) Schematic representation of the workflow from tissue sampling to data profiling. Tumour tissues were resected from nine patients with gastric adenocarcinoma (GAC) and then used for unbiased single‐cell RNA and TCR sequencing. Data analysis was further performed on each sample. (B) t‐SNE plot of 36 772 single cells colour‐coded by their major cell lineages. (C) The heatmap that shows the cosine similarity values among single cells grouped by patients. Cosine similarity value is represented by colours. The patient groups are colour‐coded according to the annotation bar on the right side. (D) t‐SNE plot of cancer cells that are colour‐coded according to its ITH level: purple: ITH‐high; green: ITH‐low. (E) The inferred score of copy number variants in two groups of patients (ITH‐high/low); *x*‐axis: ITH level; *y*‐axis: the score value of CNV. The difference of CNV score between these two groups is statistically significant (*p* < .05). (F) The violin plot illustrating the significant difference of proliferation score among higher ITH (ITH‐H) and lower ITH (ITH‐L) groups. (G) The degree of hypoxia in each group of samples was individually inferred by three different methods (Winter score, Buffa score, West score, respectively). (H) Comparison of VEGFA expression levels between ITH‐H and ITH‐L groups. (I) Violin plots showing the expression level of the three differently expressed genes related to ITH in the ITH groups in TCGA cohort. (J) Kaplan–Meier curves of TCGA‐STAD cohort. *p*‐Value was calculated using the log‐rank test; *x*‐axis: time; *y*‐axis: disease free survival. (K) Hazards ratio is significantly associated with the ITH degree (*p*‐value = .022) and stage (*p*‐value = .019) of samples in TCGA‐STAD cohort. *p*‐Values were computed by multivariate Cox regression model. The size of the blue square is proportional to hazard ratio of the variables. 95% Confidence intervals are represented as horizontal lines.

Six major cell lineages emerged, including epithelial cells, endothelial cells, fibroblasts, B cells, T cells, and myeloid cells, based on canonical markers (Figure 1B and Figure S1B–E; Table S2). Subpopulations were characterised by reclustering within each major cell type (Figure S1F,G).

Malignant epithelial cells were identified using inferCNV (Figure S2A). Evaluation of pair‐wise similarity of cancer cell transcriptomics revealed distinct levels of intratumour heterogeneity (ITH) across patients (Figure 1C and Figure S2B). Accordingly, we classified patients into higher ITH (ITH‐H) and lower ITH (ITH‐L) groups based on the median of ITH score (Supporting Information Methods), validated by the observation that ITH‐H tumours spanned multiple epithelial clusters, which were scarce in ITH‐L tumours (Figure 1D and Figure S2C,D). ITH‐H cancer cells exhibited higher levels of copy number variation (CNV), proliferation and hypoxia compared with their ITH‐L counterparts (Figure 1E–G), along with elevated *VEGFA* expression, indicating hypoxia‐driven angiogenesis (Figure 1H). Differentially expressed genes (DEGs) analysis between ITH‐H and ITH‐L groups identified an ITH signature (*PSCA*, *PPIB* and *CALR*) dividing TCGA‐STAD data into ITH‐H and ITH‐L groups based on the median ITH signature score calculated by ssGSEA (Figure 1I and Figure S2E). ITH‐H TCGA‐STADs displayed increased proliferation, hypoxia and angiogenesis compared with the ITH‐L tumours (Figure S2F–H). Furthermore, ITH correlated with inferior disease‐free survival (DFS) in TCGA‐STADs, even after adjusting for confounding factors in multivariate COX regression analysis (Figure 1J,K), suggesting ITH as an independent prognostic factor for recurrence.^3^

Considering Lauren's subtype, intestinal‐type epithelial cells exhibited higher levels of CNV (Figure S2I), consistent with previous reports.^4^ However, no association was observed between Lauren's subtype and ITH degree either in our cohort or in TCGA‐STAD data (Figure S2J,K).

In the stromal compartment, three endothelial cell types and two fibroblast types were identified. Notably, CXCL14^+^ fibroblasts, enriched in the ITH‐H tumours, displayed oncogenic potential (Figures S3 and S4, Supporting Information).

Immune cell analysis explored the immune tumour microenvironment (TME) in relation to ITH. Among myeloid cells, we identified dendritic cells, monocytes, mast cells and macrophages (Figure S5A–D). Mφ‐SPP1, a transitional state between Mφ‐FCN1 and Mφ‐APOE, expressed mixed expression of *SPP1* (a tumour‐associated macrophage marker), *ALOX5AP* (a crucial immune‐modulating lipid mediator associated with M2 macrophage polarization) and *CD163* (an M2 macrophage marker)^5^, ^6^ (Table S3; Figure 2A–C). Distinct macrophage compositions were observed between different ITH statuses or among different Lauren's subtypes (Figure S5E). Unique transcriptional factors (TFs) regulatory network activity, particularly MYC(+), was observed in Mφ‐SPP1 (Figure 2D). *MYC* was previously reported as a potent regulator in tumour‐associated macrophages (TAMs).^7^, ^8^ Consistently, we detected high expression of *MYC* and its target genes specific to Mφ‐SPP1 (Figure 2E,F and Figure S5F). Moreover, the targets of *MYC* were mainly upregulated in Mφ‐SPP1 compared with the other two cell types (Figure 2G,H). In addition to the top targets inferred by pySCENIC, we observed upregulation of *MRC1* and *PARG* in Mφ‐SPP1, which were previously reported as the targets of MYC in TAMs (Figure 2H).^8^ The above findings confirm Mφ‐SPP1 as a TAM subtype and suggest potential downstream genes involved in the TAM function. We also observed that macrophages in the ITH‐H group were mainly located at the end stage of pseudotime and exhibited an enriched anti‐inflammatory signature (Figure S5G,H, Supporting Information).

**FIGURE 2:**
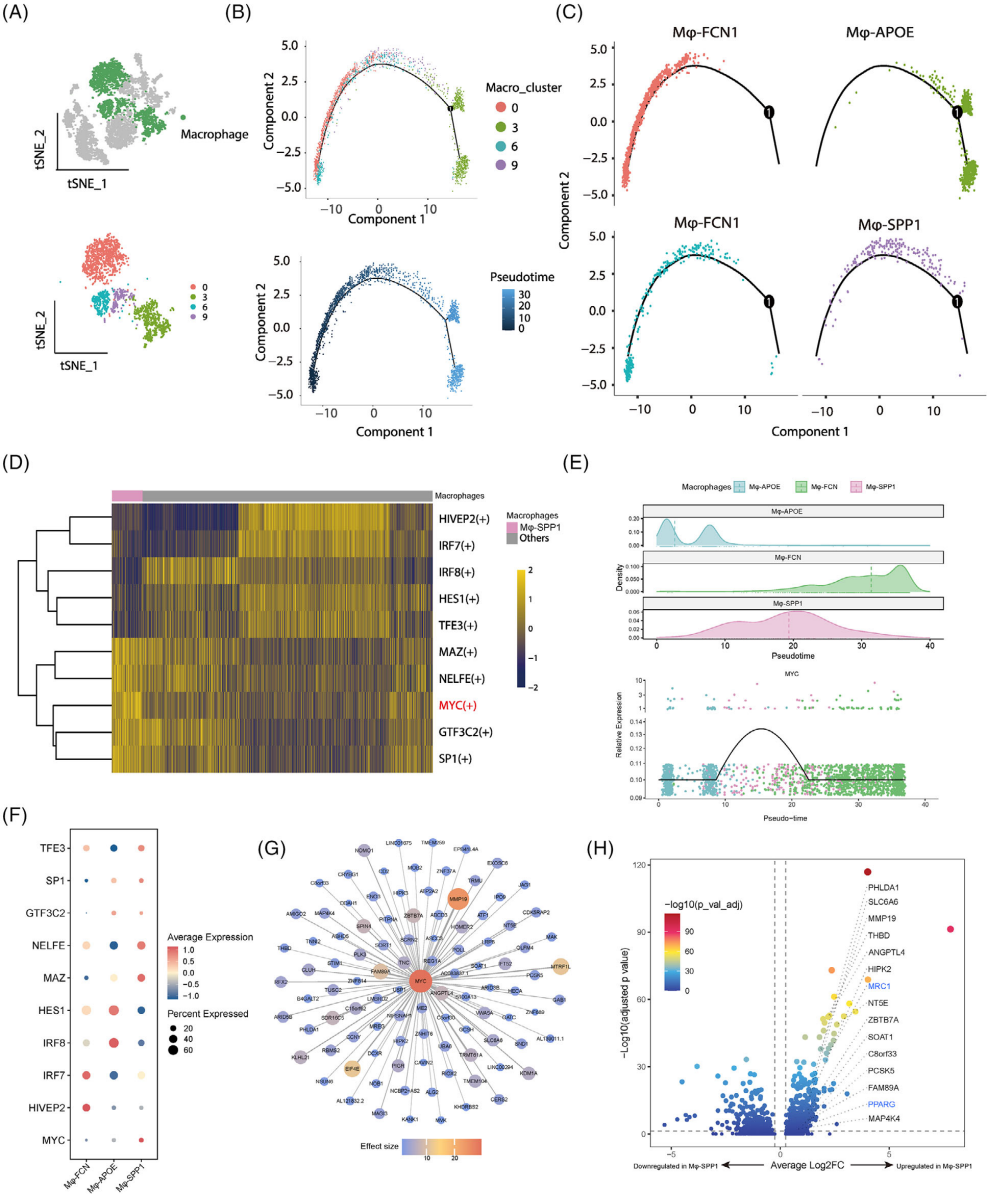
Mφ‐SPP1 represents a type of tumour‐associated macrophage. (A) t‐SNE plot shows macrophage cells (top) on four sublineages (bottom). Each sublineage is annotated and colour‐coded accordingly. (B) Unsupervised transcriptional trajectory of macrophage cells from Monocle (version 2), coloured by either cell sublineages (top) or the corresponding pseudotime (bottom). (C) The separated trajectory of each macrophage sublineage is shown. (D) Heatmap of TFs and their regulation networks (regulons) with differential activity between Mφ‐SPP1 and the other macrophage sublineages. (E) The distribution of Mφ‐SPP1, Mφ‐FCN and Mφ‐APOE and MYC expression along the pseudotime. (F) Dot plot showing the expression of the 10 TFs (D) among Mφ‐SPP1, Mφ‐APOE and Mφ‐FCN. (G) The transcriptional regulatory network showing the top 100 target genes (estimated by pySCENIC) of MYC. Colour represents the effect size. (H) Volcano plot showing DEGs between Mφ‐SPP1 and the other sublineages of macrophages. Significantly upregulated genes within the top 100 MYC targets (G) are labelled in black. Previously reported MYC targets in tumour‐associated macrophages are labelled in blue.

T‐cell analysis revealed fewer activated and more exhausted CD8 T cells in ITH‐H tumours compared with ITH‐L tumours (Figure 3A). Stratified analysis in ITH‐H and ITH‐L groups revealed more frequent state transitions between CD8 cells in the ITH‐H group by scTCR‐seq analysis (Figure 3B **,C**). The T‐cell clones with state transition showed marked expression of immunosuppressive molecules (Figure 3D). These observations further indicate an immunosuppressive TME associated with ITH‐H (Supporting Information).

**FIGURE 3:**
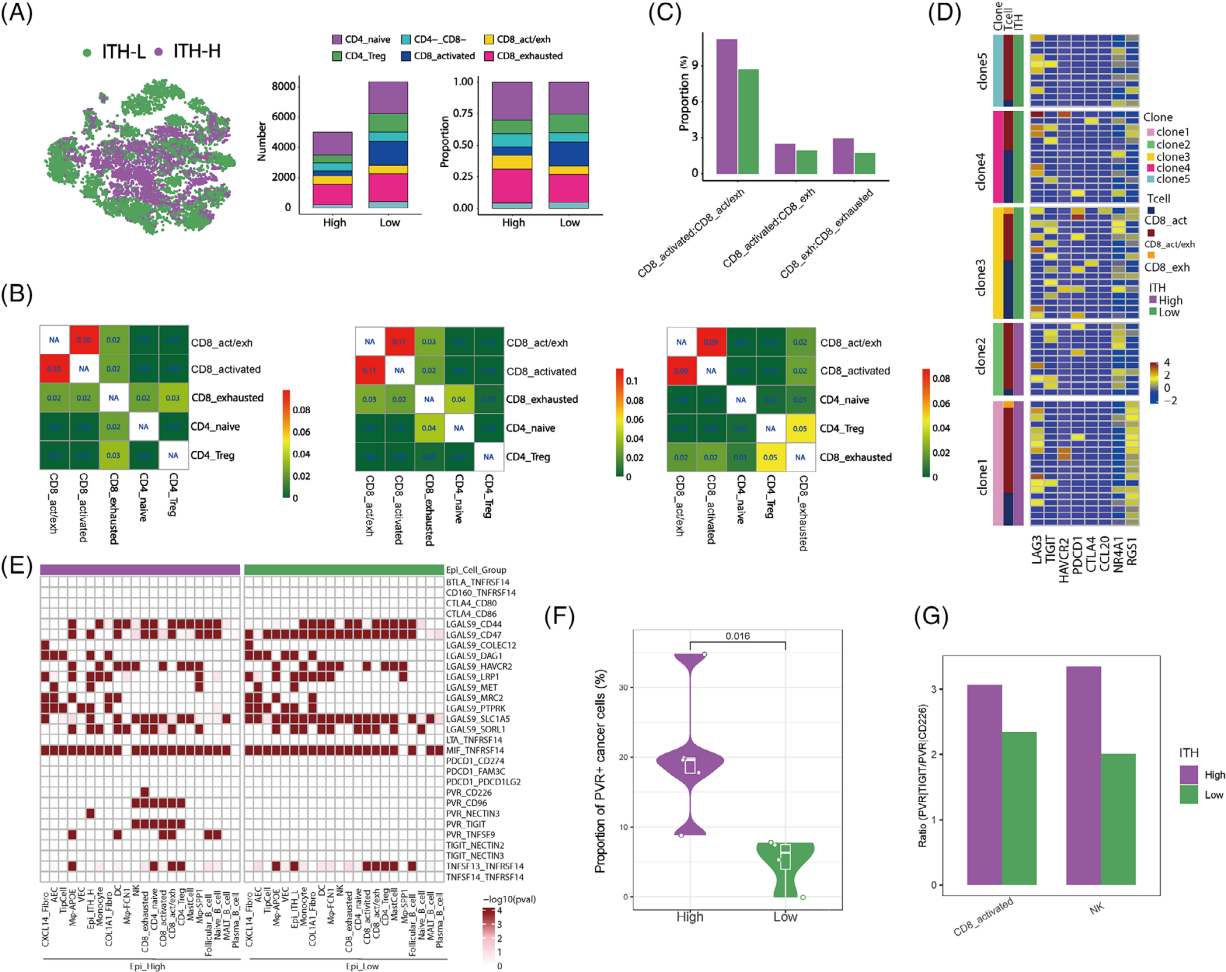
The tumours with higher intratumour heterogeneity (ITH‐H) harbour an immunosuppressive TME. (A) Left: t‐SNE plot that shows the distribution of T cells within samples with different ITH degrees. Right histograms: The number and proportion of subtypes of T cells in each ITH degree classification. (B) Heatmap presents the possibility of transformation within each pair of T‐cell lineages occurring in all the samples (left), in the ITH‐H group (middle) and in the lower ITH (ITH‐L) group (right). (C) The ability of transforming from CD8^+^ activated cell to CD8^+^ activated/exhausted cell, from CD8^+^ activated cell to CD8^+^ exhausted cell, from CD8^+^ activated/exhausted cell to CD8^+^ exhausted cell, varies between ITH‐H and ITH‐L groups. (D) Comprehensive heatmap showing the expression level of classic genes that are related to immunosuppression or immune checkpoints between T cells in terms of their sublineage, clone type and ITH degree classification. The heatmap is divided into five rectangles according to the clone type of T cells. Annotation bar is on the right side of the heatmap. (E) Heatmap shows the ligand–receptor pairs related to immune checkpoints between malignant epithelial cells and other cell lineages. Result is split into two parts according to the ITH degree classification. Significant ligand–receptor pairs are highlighted by red colour. (F) Violin plot showing the proportion of epithelial cells expressing *PVR* grouped by different ITH degrees. (G) Bar plot comparing the ratio of *PVR–TIGIT* pair to *PVR–CD226* pair between different ITH degrees in activated CD8 T cells and NK cells, respectively.

Finally, we compared the receptor–ligand pairs functioning as immune checkpoints between ITH‐H and ITH‐L groups (Supporting Information). Interestingly, we found no significant interactions between *PDCD1* and its ligands when analysing the interaction between epithelial cells and other cell populations, suggesting that anti‐PD1 therapy might not be suitable for some GACs. However, significant interaction of *PVR* and its receptors was only observed in ITH‐H tumours (Figure 3E). We also observed enrichment of *PVR* ^+^ cancer cells in the ITH‐H group compared with ITH‐L (Figure 3F). Considering the activated CD8 T cells and NK cells, along with their interactions with cancer cells through *PVR*–*TIGIT* and *PVR–CD226* interactions, the ratio of *PVR*–*TIGIT* to *PVR*–*CD226* in the ITH‐H group exceeded that in the ITH‐L group. This implies that the dominance of *TIGIT* over *CD226* was more pronounced in the ITH‐H tumours (Figure 3G), which might contribute to the immunosuppressive feature. Given that targeting *PVR* and *TIGIT* attracted increasing interest in the field of cancer immunotherapy,^9^ these findings suggest that patients with higher ITH might benefit from PVR/TIGIT inhibition treatment.

In conclusion, using a novel scoring method based on cellular similarity of transcriptional profiles, we classified GACs into different ITH groups and comprehensively analysed the associated TME heterogeneity. The study suffered limitations of small sample size and potential bias in sample selection. The presence of only one mixed GAC among the cohort hindered the accurate comparison of Lauren's subtypes. And the study only provided a cross‐sectional snapshot of the intertwining of ITH and TME, lacking over‐time observations. Nevertheless, our research suggests that ITH can serve as a prognostic marker indicative of recurrence. Immunosuppressive features such as M2 macrophage polarization, T‐cell exhaustion and interactions between certain immune checkpoint molecules are enriched in ITH‐H tumours, offering valuable insights for immunotherapeutic strategies. Longitudinal studies involving larger cohorts are essential to unravel the dynamic interaction between ITH and TME with increased statistical power.

## AUTHOR CONTRIBUTIONS

ZH Chen, HL Liu and XF Xia conceived and designed the study. J Ge, HY Zhou, MM Tang, CH Huang, X Feng and T Liu collected samples as well as clinical information. J Ge, HY Zhou, MM Tang, CH Huang, X Feng and T Liu performed the experiments. CL Zhang, J Ge, XC Zou and X Xiao analyzed the data. X Xiao, CL Zhang and J Ge wrote the manuscript. J Ge, CL Zhang, J Bai, XF Xia, HL Liu, ZH Chen and X Xiao provided intellectual discussions and ideas regarding the content of manuscript. ZH Chen, HL Liu, XF Xia and J Bai supervised the study. All authors read and approved the final manuscript.

## CONFLICT OF INTEREST STATEMENT

The authors declare no potential conflicts of interest.

## FUNDING INFORMATION

This study was supported by the National Natural Science Foundation of China (8197103463) to Zihua Chen, Hunan Province Science and Technology Innovation Plan Project (2018SK52604) ‘Key Techniques for Prevention and Treatment of Postoperative Peritoneal Recurrence and Metastasis of Gastric Cancer Based on Minimally Invasive Surgery’ to Heli Liu and Guangdong Yiyang Healthcare Charity Foundation (JZ2022014) to Jie Ge.

## Supporting information

Supporting Information

## Data Availability

The data reported in this paper have been deposited in the OMIX, China National Center for Bioinformation/Beijing Institute of Genomics, Chinese Academy of Sciences (https://ngdc.cncb.ac.cn/omix: accession no. OMIX004144).
