# Supplementary material for: Single‐cell profiling reveals tumour cell heterogeneity accompanying a pre‐malignant and immunosuppressive microenvironment in gastric adenocarcinoma
Source: Clin Transl Med. 2023 Nov 30;13(12):e1490. doi: 10.1002/ctm2.1490 (PMC10689970; doi:10.1002/ctm2.1490)
Supplement: Supplementary file 1 — Supporting Information [file CTM2-13-e1490-s001.docx]

**Supplemental Material**

# Single-cell profiling reveals tumor cell heterogeneity accompanying a pre-malignant and immunosuppressive microenvironment in gastric adenocarcinoma

Jie Ge, Xiao Xiao, Haiyan Zhou, Mimi Tang, Jing Bai, Xinchen Zou, Chunliu Zhang, Changhao Huang, Xiang Feng, Ting Liu, Xin Yi, Xuefeng Xia, Heli Liu, Zihua Chen

**Supplemental Notes**

**Supplemental Methods**

**Supplemental References**

**Supplemental Tables S1-S4**

**Supplemental Figures S1-S10**

## Supplemental Notes

### ITH-H tumors enriched for cancer-associated fibroblast recruitment

Among fibroblasts (**Figure S4A**), CXCL14+Fibroblasts, previously identified as cancer-associated fibroblasts [10-12], showed increased expression of CXCL14 in addition to canonical fibroblast markers (*COL1A1* and *COL1A2*; **Figure S4B**). The proportion of CXCL14+Fibro cells exceeded that of canonical fibroblasts in intestinal and mixed tumors (**Figure S4C**). Comparative analysis of hallmark pathways between the fibroblast types revealed that CXCL14+Fibro cells exhibited increased activity in angiogenesis, epithelial-mesenchymal transition (EMT), hypoxia, and oncogenic pathways (**Figures S4D-E**), indicating their role in promoting tumor progression. This finding aligned with a previous study in breast cancer linking CXCL14+Fibro cells to EMT and tumor invasion [13]. Moreover, CXCL14+Fibro cells constituted a higher proportion of fibroblasts in the ITH-H group (**Figure S4F**), suggesting their contribution to the more aggressive behavior of ITH-H tumors, which was validated by multiplex immunofluorescence staining (**Figure S4G**).

### **Functional differences of macrophages along the development trajectory**

Clustering the genes based on their expression along pseudotime in macrophages revealed three gene sets (GeneSet 1, 2 and 3) which respectively overrepresented different phases of the pseudotime course (**Figure S5I**). Specifically, GeneSet 1 tented to be overexpressed at the initial stage and were enriched in immune-, PD1/PDL1-, HIF-1- and ferroptosis-related pathways. GeneSet 3 were elevated at the intermediate stage and were associated with functional groups including HIF-1 signaling, glycolysis and leukocyte migration. At the end stage of the developmental path were enrichment of GeneSet 2 which was mainly associated with metabolism pathways such as oxidative phosphorylation and citrate cycle (**Figures S5I-J)**. Immune activity of GeneSet 2 was downregulated compared with GeneSet 1, although several immune-related pathways (antigen processing and Th cell differentiation) were also enriched. Taken together, immune activity decreased as the macrophages experienced the development from M1 to M2 state and tumor metabolism accelerated with the retreat of anti-tumor immunity, highlighting cellular metabolism as a critical determinant of the viability and function of immune cells.

### T cells derived from ITH-H tumors exhibited exhaustion features

T cells were the largest cell lineage identified in our dataset (**Figure 1B**). Further clustering within T cell compartment characterized CD4_naive, CD4_Treg, CD8_activated, CD8_exhausted, CD8_act/exh and NK cells according to curated markers (**Figures S6A**-**B**). CD4_Treg and CD8_activated cells were the major T cell type in diffuse GACs, while CD4_naive and CD8_exhausted cells accounted for the main fraction of T cells from intestinal type (**Figure S6C**).

The pseudotime trajectory of CD8 cells demonstrated a branching structure with CD8_activated cells enriching at the beginning stage and CD8_exhausted cells aligning at the end of both branches (**Figure S6D**). Consistent with the trajectory inference, we observed a decrease of cytotoxicity score of CD8 cells along the pseudotime path accompanied by a slight increase of exhaustion score. The cytotoxicity to exhaustion ratio reflecting the combing effect of cytotoxicity and exhaustion further proved that dysfunctional CD8 cells were enriched at the end of development (**Figure S6E**). By comparing the cytotoxicity, exhaustion and cytotoxicity to exhaustion ratio among CD8_activated, CD8_act/exh and CD8_exhausted cells, we further confirmed that they had different potential in killing target cells, and CD8_act/exh cells represented a transitional state between cytotoxicity and exhaustion (**Figure S6F**). Revealed by Monocle, the expression of cytotoxicity related genes such as *FCGR3A*, *KLRF1*, *GNLY* and *GZMA* were elevated at the early stage of development followed by the increased activity of *RGCC*, *NR4A1* at the transitional stage (**Figures S6G** and **S7A**). As reported by previous studies, *RGCC* was related to cell cycling and *NR4A1* could regulate T cell differentiation [14]*.* Therefore，the upregulation of these regulatory components suggested a continuously abating activity of CD8 cells during the development. Among the genes clustering at the end of the development, we noticed *RGS1* (**Figures S6G** and **S7A**), which played a role in prohibiting the trafficking of T cells to tumors [15]. *CCL20* was also highly expressed at the end of the development (**Figures S6G** and **S7A**), and it could mediate T cell exhaustion and M2 macrophages dependent immunosuppression together with its receptor *CCR6* [16]. Pathway analysis indicated that signaling pathways involved in immune cell functionality were prevalent along the pseudotime trajectory, suggesting the central role of CD8 cells in anti-tumor response (**Figure S7B**). The potential dependence of exhausted CD8 cells on oxidative phosphorylation was evidenced by its enrichment in GeneSet2 highly expressed at the late developmental stage (**Figures S6G and S7B**). HIF-1 signaling pathway emerged in the middle of pseudotime development, highlighting the contribution of hypoxia to immune resistance and immune suppression (**Figure S7B, GeneSet 4-5**). Therefore, multiple mechanisms contributed to the inhibitory status of CD8 cells at terminal stage of development in GACs. Distributions of CD8 cells in each Lauren’s subtype were comparative with the highest peak appearing at the end of the pseudotime (**Figure S6H**). When comparing this distribution between ITH-H and ITH-L groups, we found that although CD8 cells from both groups were enriched at the terminal stage, ITH-H seemed to harbor more dysfunctional CD8 cells (**Figure S6I**). We further confirmed the above findings by clustering the CD8 cells into 7 states and identified a large proportion of cell states belonging to the late stage as well as a small fraction of cell states at the early stage in ITH-H tumors (**Figure S6J**). Taken together, high degree of ITH was associated with lower T cells infiltration and higher T cell exhaustion.

### Clonal dynamics and phenotype transitions of T cell in ITH-H tumors

TCRs are often used as unique identifiers of T cells, and we performed scTCR-seq to track the lineage of each single T cell. After eliminating non-productive alleles (eg., out-of-frame transcripts) or low-abundance TCRs (**Figures S8A-B**), we acquired TCR data from 9,017 of 13,358 T cells. Additionally, TCR data was not available from CD4-CD8- T cells, NK cells and a fraction of CD8_activated cells (**Figure S9A**). We identified 2,350 clonal T cells and 6,667 unique T cells by assessing the shared TCR between T cells (**Figure S9B**). We further investigated TCR rearrangements to identify V-D-J usage preference and found the expected TCR segment usage bias (**Figures S8C-F**). Clonal expansion in CD8 cells was more prominent than in CD4 cells (**Figures S8G and S9C**), which was similar with a previous report [17]. When comparing the clone size among CD8 cells with different activity, we found the clonal expansion declined with the decrease of cytotoxicity and increase of exhaustion (**Figure S9D**). Additionally, the clonal expansion of different CD8 cell states varied among the Lauren’s subtypes. Specifically, CD8_activated cells from intestinal-type tumors displayed increased clonal expansion compared with those from the other subtypes. Whereas the clonality of CD8_act/exh cells from the diffuse group was higher than those in the other groups. We did not observe evident clonal expansion of exhausted CD8 cells in intestinal or diffuse types, which was instead noticeable in the single mixed-type GAC (**Figure S9E**). These findings highlighted the distinction of immune environment across different morphological phenotypes. Next, we compared the clonality of each cell state between ITH-H and ITH-L tumors and identified increased clonality of CD8_activated and CD8_exhausted cells in ITH-H group (**Figure S9F**). Generally, ITH-H was associated with stronger cytotoxicity.

We also detected phenotype transition among T cells by measuring the shared fraction of clonotypes as reported previously [17]. The results revealed common overlap between CD8 cells, indicating the plasticity of the cell fate of CD8 cells. On the contrary, we found no state transition between CD4 cell lineages. Moreover, a minimal overlap between CD8_exhausted and CD4_Treg was identified, suggestive of responding to the same antigen (**Figure 3F**). To investigate the difference of phenotype transition between tumors with different degree of ITH, we performed stratified analysis in ITH-H and ITH-L groups and found that state transition between CD8 cells was more frequent in ITH-H group (**Figures 3F-G**). To explain the underlying mechanism of phenotype transition, we further evaluated the expression of immune checkpoint genes in CD8 cell clone types with phenotype transitions. Several inhibitory molecules were highly expressed in these clones irrespective of the ITH level, histological subtypes or CD8 cell subtypes, implying the potential immunosuppression contributing to phenotype transition (**Figure S9G**). In summary, T cells in the microenvironment of ITH-H tumors were predominantly exhaustive or dysfunctional cells.

### Intercellular network uncovered divergent crosstalk in GAC

In order to unveil the interplay among different cell populations, we performed cell-cell interaction analysis based on the receptor-ligand interactions. First, we focused on the Mφ-SPP1 we previously identified as a transitional state of macrophage. We found broad interactions between this cell type and others which tended to be intensified in ITH-H tumors (**Figures S10A**-**B**). Notably, among the top 20 significant receptor-ligand pairs (**Figure S10C**), *SPP1*-*CD44* could promote M2 polarization of macrophage [18]; and *SIRPA*-*CD47* was reported as an inhibitor of phagocytosis and a potential ICI in anticancer therapy [19]. These findings further supported our previous observation that Mφ-SPP1 were associated with M2 polarization.

We next investigated the interaction between malignant epithelial cells and other cell lineages regarding the ITH degree and found that cancer cells had more active crosstalk with other cell populations in ITH-H group (**Figures S10D**-**E**). Of note, although most interactions of chemokines and their receptors were weak, the interaction between malignant cells and monocytes mediated by *CXCL1-CXCR2*，*CXCL2-CXCR2* and *CXCL3-CXCR1* was conspicuous in ITH-H group (**Figure S10F**). According to previous literature, *CXCL1-CXCR2* and *CXCL2-CXCR2* were involved in progression of gastric cancers [20]. Additionally, *CXCL3-CXCR2* could promote recruitment of myeloid-derived suppressor cells in bladder cancers [21]. Therefore, these chemokine signaling pathways might play suppressive role in shaping the immune environment of ITH-H tumors. Taken together, the inter-cellular interactions suggest a close relationship between immune cell dynamics and molecular features of malignant cells, which may shed light on the therapeutic strategy in GAC.

## Supplemental Methods

### Sample collection and tissue processing

All procedures were performed regarding ethical regulations of Ethics Committee of Xiangya Hospital of Central South University and in accordance with Declaration of Helsinki. Written informed consent was obtained from all patients enrolled in this study. Eight surgical and one gastroscopic biopsies specimens were obtained from a cohort of treatment-naïve GAC patients consisting of 5 diffuse, 3 intestinal and 1 mixed type. The pathological diagnosis was confirmed by two independent expert pathologists. No evidence of HP or EBV infection was found in our specimens. Detailed clinical characteristics are provided in Table S1. The fresh tissues were immediately stored in ice-cold RPMI-1640 (Gibco) supplemented with 10% fetal bovine serum (FBS, Gibco) after resection and gastroscopic biopsy. Tumor tissues were cut into small pieces (1-2 mm3) followed by enzymatic digestion using MACS tumor Dissociation Kit (Miltenyi Biotec) at 37°C with mild agitation. To obtain single cell suspension, the dissociated cells were passed through a sterile 70μm mesh (BD) and centrifuged at 400 g and 4°C for 10 minutes. Cell pellets were collected after discarding the supernatant and resuspended in red blood cell (RBC) lysis buffer (Roche) for removing RBC. Cells were washed with PBS and subsequently resuspended in PBS supplemented with 1% FBS for further processing.

### Library preparation and scRNA-seq

After adjusting the cell concentration to ~100,000/ml and loading cells onto the cartridge (10× Genomics), we used Chromium Single Cell 5′Library & Gel Bead Kit and Chromium Single Cell V(D)J Enrichment Kit (10× Genomics) to prepare the cDNA libraries and TCR VDJ libraries respectively. All procedures were performed according to the manufacturer’s instructions. The libraries were sequenced on an Illumina nova-seq 6000 platform with 150 bp paired-end read.

### Data processing

CellRanger (10× Genomics) was used to analyze the sequencing data. In brief, the gene expression data was mapped to GRCh38 human reference genome. After correction of batch effect with Harmony, we used Seurat 3.2.2 to perform the downstream pipeline. Cells with fewer than 200 genes or 800 UMIs were removed. Additionally, cells with mitochondrial RNA content greater than 25% or RBC RNA over 2% were excluded. We identified doublets with DoubletFinder and excluded them from subsequent analysis. Finally, we acquired 36,772 cells in total with an average of 4310 cells per each sample (range, 1,704~7,526). On average, 1,722 genes and 5,566 unique transcripts per cell were obtained.

The TCR data was aligned, assembled and annotated using CellRanger VDJ (10× Genomics). Only the high-confident, productive TCR clones with UMIs ≥ 2 were remained and assigned with a cell barcode, which served as the reference for integrating scRNA-seq data and scTCR-seq data for further analysis. Each cell was assigned with at least one productive TCR α or one β chains for subsequent analysis. After aforementioned quality control pipeline, 9,017 cells with TCR and RNA information were identified. For cells with only one TCR α and one β chain, cells with identical TCR α or β chains were defined as a T cell clone. Overall, we detected 7393 T cell clone in total. If two or more cells had an identical T cell clone, this TCR clonotype was identified as clonal otherwise unique. The clonal size (range, 1~58) was defined as the number of cells belonging to the same clonotype. We identified shared motifs in the CDR3 sequence and used the overlapping cell fraction to evaluate cell state transition as reported previously [17]. If one cell had an α chain composed of V segment TRAV1-2 and one of the following J segments (TRAJ33, TRAJ20 and TRAJ12), the cell was classified as a mucosal associated invariant T cell which was excluded from our analysis.

### Dimension reduction and unsupervised clustering

We generated normalized expression matrices using the function *NormalizeData* and *ScaleData* in Seurat 3.2.2. To reduce the data dimension, we performed principal component analysis with top 2000 variable genes characterized by *FindVariable* function. The first 50 principal components were used as input to perform clustering based on shared nearest neighbor (SNN) algorithm at a resolution of 2. Further dimensionality reduction was performed by unsupervised t-distributed stochastic neighborhood embedding (t-SNE) analysis using *RunTSNE* function in Seurat. *DimPlot* function was applied to visualize the clustering results. Six major cell types were identified by canonical markers (**Table S2**).

### Multiplex immunofluorescent staining

Multiplex immunofluorescent staining (mIF) was performed to validate the existence of CXCL14+Fibro cells. Briefly, Standard histological methods were used to slice paraffin-embedded tissues into 4 µm slides. After dewaxing and antigen retrieval, the sections were stained with Akoya Opal^TM^ staining kit, including CXCL14 (Abcam, ab46010, Opal690) and COL1A1 (cell signaling technology, CST#39952S, Opal620) according to the manufacturer's protocol. The cell nuclei were stained with 4’,6-diamidino-2-phenylindole dihydrochloride (DAPI). The stained slides were finally photographed using a laser scanning confocal microscope from Olympus and analyzed with the CellSens Dimension Software from Olympus.

### Cell type identification

Major clusters were further subjected to reclustering to identify subclusters using methods described above with the following parameters: nfeatures = 2000, dims = 1:20, resolution = 0.7. ﻿We used the function *FindAllMarkers* in Seurat 3.2.2 to identify differently expressed genes (DEGs) between different cell types with the following threshold: |log2 fold change| ≥ 0.25 and P value < 0.05 (Wilcoxon Rank Sum test). Within each lineage, an iterative process was applied to remove putative doublet clusters, if any, and the remaining cells were reclustered. Putative doublets were identified by double positive expressions of the canonical marker genes of 6 major cell types characterized above. Clusters were annotated based on the normalized expression of the curated marker genes. Details of the cell types and corresponding marker genes are listed in **Table S2**. Additionally, we defined the subset of macrophage by the top 50 marker genes in this subcluster (**Table S3**).

### CNV analysis from scRNA-seq data

We used inferCNV to identify large-scale chromosomal aberration from scRNA-seq data. Fibroblast cells and endothelial cells were set as reference cells with the following parameters: cutoff=0.1, hcluster_method=“ward.D2”, HMM=T, denoise = TRUE. The expression pattern of epithelial cells was compared with the reference to differentiate malignant cells which exhibited copy number alteration. We used quadratic sum of (state -3)/2 to evaluate CNV score of each cell with changing all sated 6 (if any) to 5 in HMM prediction result.

### ITH calculation

Enlightened by previous reports[22, 23], we calculated ITH by calculating pairwise distance of malignant cells (measured by Cosine Similarity) based on the relative expression of genes. Interquartile range (IQR) of the distribution of all pairs’ distance in each sample was used to define ITH.

### Generation of ITH-associated gene signature

To identify a gene expression signature that is clinically applicable, we compared gene expression profiles of tumor cell between ITH-H and ITH-L groups with *FindMarkers* in Seurat 3.3.2 and generated DEGs. Based on these DEGs, three genes (*PSCA*, *PPIB* and *CALR*) highly expressed in ITH-H group were defined as ITH-associated gene signature with the following cutoff: log2 expression fold change > 0.25, adjusted p value < 0.05 and the gap between pt.1 and pt.2 > 0.25.

### Definition of cell scores for a functional signature with scRNA-seq data

We used the average expression measured by log2 (TPM + 1) of a given set of signature genes to calculate a corresponding score. Three independent hypoxia- associated gene sets were adopted to evaluate the hypoxia degree [24-26]. In addition, 23 proliferation-related genes were used to define proliferation score. We assigned a score for the pro-/anti-inflammatory using previously defined gene sets [27]. Exhaustion and activation score were calculated by exhaustion signature genes and activation signature genes defined previously [28]. The details of gene sets used for measuring a functional signature are illustrated in **Table S4.** CD4-CD8- cells were excluded from the analysis. MALANT1+ myeloid cells were not involved, since we could not annotate these cells by the canonical marker genes of the myeloid cell subtype. The top 50 marker genes in the subset of macrophage are listed in **Table S3**.

### Gene signature analysis in TCGA data

We used single-sample Gene Set Enrichment Analysis (ssGSEA) to assess signature score based on a given gene set (as above mentioned) in TCGA bulk RNA profiling data.

### Survival analysis

The TCGA-STAD (stomach adenocarcinoma) dataset (downloaded from http://xena.ucsc.edu/) was extracted to evaluate the association of ITH related gene signature with disease-free survival (DFS). The mean expression of the genes included in the ITH gene set was calculated for each sample and every dataset was divided into high and low expression groups using the median value as the cutoff. The survival analysis was carried out using R function *survfit* from survival package*.* Kaplan-Meier survival curves were plotted, and log-rank test was performed using R function *ggsurvplot* from survminer package. Multivariate COX regression was performed to adjust for confounders, and the hazard ratio (HR) and 95% confidence interval (CI) were calculated.

### Cell developmental trajectory

The pseudo-time of cell lineage development was analyzed by Monocle2 [29]. We used the *differentialGeneTest* function to derive DEGs from each cluster and genes with q-value<1e-5 were assessed to order the cells in pseudotime analysis. After the cell trajectories were constructed, DEGs along the pseudotime were detected using the *differentialGeneTest* function. Subsequently, *plot_pseudotime_heatmap* and *plot_cell_trajectory* were performed to visualize cells order in pseudotime progression.

### Cell-cell interaction analysis

Cell-cell interactions between different cell lineages were inferred by CellPhoneDB based on the ligand-receptor pairs [30]. Specific expression of a receptor by one cell type and a corresponding ligand by another cell type was identified to reflect the potential interaction between cell types. The *igraph* package in R was used to visualize the interaction network among various cell types. Each cell type was considered as a node and the interactions between two cell types was taken as an edge with its width proportional to the number of the interactions. The difference of interactions between ITH-H group and ITH-L groups (Delta (ITH-H-ITH-L)) was calculated as the number of interactions in ITH-H group minus that in ITH-L group regarding every pair of cell types of interest. We next analyzed important intercellular signals such as chemokines and immune checkpoints between cell types. The average expression values of the individual ligand-receptor complex in the corresponding interacting pairs of cell types were calculated to measure the interaction score.

### Statistics

Statistical analysis was performed using R (version 4.0.3). Wilcoxon rank-sum test was used to compare groups of variables. Kruskal-Wallis test was used to compare three or more groups of variables. A *p* value < 0.05 was considered as statistical significance.

## Supplemental References

10. Augsten M, Sjoberg E, Frings O, Vorrink SU, Frijhoff J, Olsson E, Borg A, Ostman A: **Cancer-associated fibroblasts expressing CXCL14 rely upon NOS1-derived nitric oxide signaling for their tumor-supporting properties**. *Cancer Res* 2014, **74**(11):2999-3010.

11. Zhang Q, Zhou N, Wang W, Zhou S: **A Novel Autocrine CXCL14/ACKR2 Axis: The Achilles' Heel of Cancer Metastasis?** *Clin Cancer Res* 2019, **25**(12):3476-3478.

12. Gowhari Shabgah A, Haleem Al-Qaim Z, Markov A, Valerievich Yumashev A, Ezzatifar F, Ahmadi M, Mohammad Gheibihayat S, Gholizadeh Navashenaq J: **Chemokine CXCL14; a double-edged sword in cancer development**. *Int Immunopharmacol* 2021, **97**:107681.

13. Sjoberg E, Meyrath M, Milde L, Herrera M, Lovrot J, Hagerstrand D, Frings O, Bartish M, Rolny C, Sonnhammer E *et al*: **A Novel ACKR2-Dependent Role of Fibroblast-Derived CXCL14 in Epithelial-to-Mesenchymal Transition and Metastasis of Breast Cancer**. *Clin Cancer Res* 2019, **25**(12):3702-3717.

14. Liu X, Wang Y, Lu H, Li J, Yan X, Xiao M, Hao J, Alekseev A, Khong H, Chen T *et al*: **Genome-wide analysis identifies NR4A1 as a key mediator of T cell dysfunction**. *Nature* 2019, **567**(7749):525-529.

15. Huang D, Chen X, Zeng X, Lao L, Li J, Xing Y, Lu Y, Ouyang Q, Chen J, Yang L *et al*: **Targeting regulator of G protein signaling 1 in tumor-specific T cells enhances their trafficking to breast cancer**. *Nat Immunol* 2021, **22**(7):865-879.

16. Palena C, Gulley JL: **A rare insight into the immunosuppressive landscape of prostate cancer bone metastases**. *Cancer Cell* 2021, **39**(11):1450-1452.

17. Yost KE, Satpathy AT, Wells DK, Qi Y, Wang C, Kageyama R, McNamara KL, Granja JM, Sarin KY, Brown RA *et al*: **Clonal replacement of tumor-specific T cells following PD-1 blockade**. *Nat Med* 2019, **25**(8):1251-1259.

18. Liu L, Zhang R, Deng J, Dai X, Zhu X, Fu Q, Zhang H, Tong Z, Zhao P, Fang W *et al*: **Construction of TME and Identification of crosstalk between malignant cells and macrophages by SPP1 in hepatocellular carcinoma**. *Cancer Immunol Immunother* 2021.

19. Veillette A, Chen J: **SIRPalpha-CD47 Immune Checkpoint Blockade in Anticancer Therapy**. *Trends Immunol* 2018, **39**(3):173-184.

20. Yamamoto Y, Kuroda K, Sera T, Sugimoto A, Kushiyama S, Nishimura S, Togano S, Okuno T, Yoshii M, Tamura T *et al*: **The Clinicopathological Significance of the CXCR2 Ligands, CXCL1, CXCL2, CXCL3, CXCL5, CXCL6, CXCL7, and CXCL8 in Gastric Cancer**. *Anticancer Res* 2019, **39**(12):6645-6652.

21. Zhang H, Ye YL, Li MX, Ye SB, Huang WR, Cai TT, He J, Peng JY, Duan TH, Cui J *et al*: **CXCL2/MIF-CXCR2 signaling promotes the recruitment of myeloid-derived suppressor cells and is correlated with prognosis in bladder cancer**. *Oncogene* 2017, **36**(15):2095-2104.

22. Ma L, Hernandez MO, Zhao Y, Mehta M, Tran B, Kelly M, Rae Z, Hernandez JM, Davis JL, Martin SP *et al*: **Tumor Cell Biodiversity Drives Microenvironmental Reprogramming in Liver Cancer**. *Cancer Cell* 2019, **36**(4):418-430 e416.

23. Wu F, Fan J, He Y, Xiong A, Yu J, Li Y, Zhang Y, Zhao W, Zhou F, Li W *et al*: **Single-cell profiling of tumor heterogeneity and the microenvironment in advanced non-small cell lung cancer**. *Nat Commun* 2021, **12**(1):2540.

24. Buffa FM, Harris AL, West CM, Miller CJ: **Large meta-analysis of multiple cancers reveals a common, compact and highly prognostic hypoxia metagene**. *Br J Cancer* 2010, **102**(2):428-435.

25. Winter SC, Buffa FM, Silva P, Miller C, Valentine HR, Turley H, Shah KA, Cox GJ, Corbridge RJ, Homer JJ *et al*: **Relation of a hypoxia metagene derived from head and neck cancer to prognosis of multiple cancers**. *Cancer Res* 2007, **67**(7):3441-3449.

26. Eustace A, Mani N, Span PN, Irlam JJ, Taylor J, Betts GN, Denley H, Miller CJ, Homer JJ, Rojas AM *et al*: **A 26-gene hypoxia signature predicts benefit from hypoxia-modifying therapy in laryngeal cancer but not bladder cancer**. *Clin Cancer Res* 2013, **19**(17):4879-4888.

27. Sun Y, Wu L, Zhong Y, Zhou K, Hou Y, Wang Z, Zhang Z, Xie J, Wang C, Chen D *et al*: **Single-cell landscape of the ecosystem in early-relapse hepatocellular carcinoma**. *Cell* 2021, **184**(2):404-421 e416.

28. Kim N, Kim HK, Lee K, Hong Y, Cho JH, Choi JW, Lee JI, Suh YL, Ku BM, Eum HH *et al*: **Single-cell RNA sequencing demonstrates the molecular and cellular reprogramming of metastatic lung adenocarcinoma**. *Nat Commun* 2020, **11**(1):2285.

29. Qiu X, Mao Q, Tang Y, Wang L, Chawla R, Pliner HA, Trapnell C: **Reversed graph embedding resolves complex single-cell trajectories**. *Nat Methods* 2017, **14**(10):979-982.

30. Efremova M, Vento-Tormo M, Teichmann SA, Vento-Tormo R: **CellPhoneDB: inferring cell-cell communication from combined expression of multi-subunit ligand-receptor complexes**. *Nat Protoc* 2020, **15**(4):1484-1506.

## Supplemental Tables

### Table S1 Clinical Information of 9 gastric adenocarcinoma patients

| **PatientID** | **Sex** | **Age** | **Pathology** | **Lauren classification** | **TNM stage** |
| --- | --- | --- | --- | --- | --- |
| WA9 | female | 28 | Low Differentiated Adenocarcinoma | Diffuse | pT3N0M0 |
| WA2 | male | 67 | Low Differentiated Adenocarcinoma | Diffuse | pT3N0M0 |
| WA4 | male | 46 | Low Differentiated Adenocarcinoma | Diffuse | pT1bN2M0 |
| WA6 | female | 66 | Low Differentiated Adenocarcinoma | Diffuse | pT3N3aM0 |
| WA12 | female | 53 | Low Differentiated Adenocarcinoma(Linitis Plastica) | Diffuse | pT3N3bM1 |
| WA10 | male | 51 | Differentiated Adenocarcinoma | Intestinal | pT1bN0M0 |
| WA3 | male | 58 | Differentiated Adenocarcinoma | Intestinal | pT3N2M0 |
| WA5 | female | 55 | Differentiated Adenocarcinoma | Intestinal | pT3N0M0 |
| WA8 | male | 64 | Differentiated Adenocarcinoma | Mix | pT3N2M0 |

### Table S2 Selected marker genes for annotating cell types

| **Clusters** | **Cluster type** | **Selected makers** |
| --- | --- | --- |
| Epithelial Cell | Main clusters | EPCAM；KRT19；KRT18 |
| T Cell | Main clusters | CD3G，CD3E |
| B Cell | Main clusters | CD79A |
| Endothelial Cell | Main clusters | VWF |
| Fibroblast | Main clusters | COL1A1；COL1A2 |
| Myeloid Cell | Main clusters | CD14；CSF1R；TPSAB1 |
| VEC | Sub-clusters | VWF;ACKR1 |
| AEC | Sub-clusters | GJA5 |
| TipCell | Sub-clusters | KCNE3;ANGPT2;APLN;ESM1 |
| COL1A1+Fibro | Sub-clusters | COL1A1；COL1A2 |
| CXCL14+Fibro | Sub-clusters | COL1A1; COL1A2; CXCL14 |
| Follicular_B_cell | Sub-clusters | MS4A1;CD79A |
| MALT_B_Cell | Sub-clusters | JCHAIN; CD79A |
| Naïve B cell | Sub-clusters | PTPRC;BATF;IL7R |
| Plasma_B_Cell | Sub-clusters | IGKC; IGHM |
| DC | Sub-clusters | CD1C;CD1E;IDO1;CD40;CLEC10A;FCGR2B;LAMP3 |
| Macophage | Sub-clusters | CD163;SPP1;MACRO;FCN1;C1QA;C1QB;APOE;IL1A;IL1B |
| Mast Cell | Sub-clusters | TPSB2;TPSAB1;MS4A2;CSF1;CPA3 |
| Monocyte | Sub-clusters | S100A12;CD14;APOBEC3A;MS4A6A;CLEC12A |
| CD4 Naïve Cell | Sub-clusters | CD4;LEF1;CCR7;TCF7 |
| CD4 Treg Cell | Sub-clusters | CD4;FOXP3;CTLA4 |
| CD8_activited | Sub-clusters | CD8A;CD8B;KLRC3;FCGR3A;IFNG |
| CD8_exhausted | Sub-clusters | CD8A;CD8B;PDCD1 |
| CD8_activited/exhausted | Sub-clusters | CD8A;CD8B;GZMK;IFNG;PDCD1 |
| NK | Sub-clusters | KLRC1;KLRC3 |

### Table S3 Top 50 Marker genes in macrophage subset

| Genes | p_val | avg_logFC | pct.1 | pct.2 | p_val_adj | CellType |
| --- | --- | --- | --- | --- | --- | --- |
| APOE | 1.25E-292 | 3.895513077 | 0.784 | 0.057 | 2.89E-288 | Mφ-APOE |
| C1QC | 0 | 3.7893696 | 0.93 | 0.046 | 0 | Mφ-APOE |
| C1QB | 0 | 3.775572375 | 0.917 | 0.043 | 0 | Mφ-APOE |
| C1QA | 0 | 3.674462895 | 0.936 | 0.057 | 0 | Mφ-APOE |
| APOC1 | 1.26E-255 | 3.182849924 | 0.768 | 0.079 | 2.89E-251 | Mφ-APOE |
| SELENOP | 5.34E-204 | 2.809533376 | 0.525 | 0.008 | 1.23E-199 | Mφ-APOE |
| MMP12 | 4.30E-101 | 2.14489804 | 0.297 | 0.009 | 9.90E-97 | Mφ-APOE |
| GPNMB | 4.12E-251 | 2.053136342 | 0.725 | 0.062 | 9.49E-247 | Mφ-APOE |
| SLC40A1 | 3.06E-233 | 2.01589382 | 0.587 | 0.008 | 7.04E-229 | Mφ-APOE |
| RGS1 | 2.88E-188 | 1.886490885 | 0.747 | 0.161 | 6.64E-184 | Mφ-APOE |
| ACP5 | 2.16E-175 | 1.765133941 | 0.861 | 0.356 | 4.97E-171 | Mφ-APOE |
| PLD3 | 1.13E-198 | 1.742931848 | 0.839 | 0.292 | 2.59E-194 | Mφ-APOE |
| HLA-DQA1 | 3.85E-193 | 1.720260806 | 0.929 | 0.487 | 8.87E-189 | Mφ-APOE |
| CTSZ | 1.22E-221 | 1.711570946 | 0.941 | 0.573 | 2.81E-217 | Mφ-APOE |
| RNASE1 | 7.70E-54 | 1.69086167 | 0.454 | 0.172 | 1.77E-49 | Mφ-APOE |
| HLA-DQB1 | 2.16E-180 | 1.644891532 | 0.922 | 0.609 | 4.97E-176 | Mφ-APOE |
| CTSD | 3.21E-176 | 1.634530697 | 0.95 | 0.588 | 7.39E-172 | Mφ-APOE |
| TREM2 | 6.96E-204 | 1.633158461 | 0.627 | 0.051 | 1.60E-199 | Mφ-APOE |
| HLA-DPB1 | 1.07E-203 | 1.60213208 | 0.975 | 0.764 | 2.47E-199 | Mφ-APOE |
| MS4A6A | 7.37E-180 | 1.589907336 | 0.888 | 0.438 | 1.70E-175 | Mφ-APOE |
| LGMN | 1.50E-136 | 1.572320029 | 0.833 | 0.42 | 3.45E-132 | Mφ-APOE |
| HLA-DPA1 | 2.80E-190 | 1.544552918 | 0.978 | 0.793 | 6.45E-186 | Mφ-APOE |
| A2M | 7.53E-230 | 1.492041915 | 0.664 | 0.044 | 1.73E-225 | Mφ-APOE |
| CD74 | 4.56E-217 | 1.466122107 | 1 | 0.972 | 1.05E-212 | Mφ-APOE |
| CCL18 | 4.37E-52 | 1.461796266 | 0.259 | 0.044 | 1.01E-47 | Mφ-APOE |
| TMEM176B | 1.01E-179 | 1.451644116 | 0.877 | 0.394 | 2.32E-175 | Mφ-APOE |
| MMP9 | 4.87E-67 | 1.434377678 | 0.435 | 0.118 | 1.12E-62 | Mφ-APOE |
| TMEM176A | 6.65E-190 | 1.431854555 | 0.834 | 0.248 | 1.53E-185 | Mφ-APOE |
| HLA-DMB | 9.67E-206 | 1.424135304 | 0.842 | 0.264 | 2.23E-201 | Mφ-APOE |
| LIPA | 1.76E-163 | 1.414672525 | 0.744 | 0.215 | 4.06E-159 | Mφ-APOE |
| TUBA1B | 1.49E-167 | 1.370226207 | 0.911 | 0.545 | 3.43E-163 | Mφ-APOE |
| DAB2 | 7.12E-192 | 1.358503584 | 0.772 | 0.157 | 1.64E-187 | Mφ-APOE |
| HLA-DMA | 1.63E-193 | 1.333017686 | 0.928 | 0.568 | 3.75E-189 | Mφ-APOE |
| MS4A4A | 1.28E-195 | 1.330159153 | 0.777 | 0.174 | 2.96E-191 | Mφ-APOE |
| CCL24 | 9.87E-66 | 1.321386323 | 0.306 | 0.049 | 2.27E-61 | Mφ-APOE |
| DNASE1L3 | 1.07E-91 | 1.316484451 | 0.247 | 0.001 | 2.45E-87 | Mφ-APOE |
| ITM2B | 8.10E-185 | 1.313142965 | 0.963 | 0.829 | 1.86E-180 | Mφ-APOE |
| PLTP | 1.81E-189 | 1.273417088 | 0.525 | 0.018 | 4.16E-185 | Mφ-APOE |
| HLA-DRB1 | 2.71E-161 | 1.255697838 | 0.993 | 0.935 | 6.25E-157 | Mφ-APOE |
| GRN | 5.03E-200 | 1.250858466 | 0.959 | 0.771 | 1.16E-195 | Mφ-APOE |
| FOLR2 | 3.62E-106 | 1.246589247 | 0.43 | 0.061 | 8.33E-102 | Mφ-APOE |
| CTSC | 3.35E-148 | 1.233338383 | 0.932 | 0.659 | 7.71E-144 | Mφ-APOE |
| YWHAH | 2.56E-161 | 1.218996975 | 0.775 | 0.22 | 5.90E-157 | Mφ-APOE |
| MS4A7 | 1.80E-145 | 1.211429477 | 0.861 | 0.397 | 4.13E-141 | Mφ-APOE |
| FABP5 | 8.54E-81 | 1.207470106 | 0.714 | 0.324 | 1.97E-76 | Mφ-APOE |
| CST3 | 1.29E-175 | 1.186743146 | 0.99 | 0.945 | 2.98E-171 | Mφ-APOE |
| ARL4C | 3.35E-159 | 1.182632178 | 0.627 | 0.094 | 7.71E-155 | Mφ-APOE |
| FGL2 | 2.51E-158 | 1.181058217 | 0.719 | 0.173 | 5.78E-154 | Mφ-APOE |
| AKR1B1 | 3.52E-198 | 1.13671855 | 0.729 | 0.119 | 8.12E-194 | Mφ-APOE |
| TNIP3 | 7.24E-200 | 2.680090859 | 0.72 | 0.08 | 1.67E-195 | Mφ-FCN1 |
| IL1RN | 4.86E-229 | 2.525366296 | 0.909 | 0.393 | 1.12E-224 | Mφ-FCN1 |
| PTGS2 | 1.61E-224 | 2.230282904 | 0.87 | 0.317 | 3.70E-220 | Mφ-FCN1 |
| CCL4L2 | 1.72E-103 | 1.78638952 | 0.85 | 0.578 | 3.96E-99 | Mφ-FCN1 |
| IL1B | 5.48E-243 | 1.748425053 | 0.984 | 0.643 | 1.26E-238 | Mφ-FCN1 |
| IL1A | 6.24E-147 | 1.612962964 | 0.703 | 0.177 | 1.44E-142 | Mφ-FCN1 |
| CCL3L1 | 1.04E-128 | 1.606927521 | 0.878 | 0.587 | 2.40E-124 | Mφ-FCN1 |
| IL6 | 2.68E-58 | 1.604784325 | 0.435 | 0.141 | 6.17E-54 | Mφ-FCN1 |
| FCN1 | 4.85E-213 | 1.576480332 | 0.88 | 0.21 | 1.12E-208 | Mφ-FCN1 |
| TIMP1 | 2.42E-220 | 1.561721355 | 0.991 | 0.867 | 5.58E-216 | Mφ-FCN1 |
| AQP9 | 1.91E-213 | 1.558392491 | 0.871 | 0.242 | 4.39E-209 | Mφ-FCN1 |
| S100A8 | 8.55E-162 | 1.549526201 | 0.934 | 0.434 | 1.97E-157 | Mφ-FCN1 |
| SLC2A3 | 1.06E-210 | 1.524572578 | 0.944 | 0.571 | 2.44E-206 | Mφ-FCN1 |
| MIR3945HG | 5.91E-133 | 1.474695015 | 0.669 | 0.171 | 1.36E-128 | Mφ-FCN1 |
| SOD2 | 3.63E-250 | 1.401656567 | 0.994 | 0.899 | 8.36E-246 | Mφ-FCN1 |
| EREG | 1.56E-141 | 1.391810549 | 0.81 | 0.288 | 3.58E-137 | Mφ-FCN1 |
| CD300E | 1.31E-164 | 1.391682124 | 0.809 | 0.225 | 3.00E-160 | Mφ-FCN1 |
| CCL20 | 8.03E-96 | 1.37826129 | 0.747 | 0.322 | 1.85E-91 | Mφ-FCN1 |
| TNIP1 | 2.75E-181 | 1.374364567 | 0.855 | 0.457 | 6.33E-177 | Mφ-FCN1 |
| SERPINB9 | 2.43E-188 | 1.352238893 | 0.866 | 0.46 | 5.60E-184 | Mφ-FCN1 |
| PLAUR | 2.59E-216 | 1.350648362 | 0.993 | 0.846 | 5.96E-212 | Mφ-FCN1 |
| CCL4 | 6.54E-97 | 1.335627675 | 0.898 | 0.684 | 1.51E-92 | Mφ-FCN1 |
| CCL3 | 3.04E-129 | 1.316057888 | 0.939 | 0.755 | 7.00E-125 | Mφ-FCN1 |
| BCL2A1 | 4.83E-206 | 1.309685959 | 0.967 | 0.678 | 1.11E-201 | Mφ-FCN1 |
| TNFAIP6 | 2.38E-132 | 1.288423738 | 0.69 | 0.172 | 5.48E-128 | Mφ-FCN1 |
| S100A9 | 5.50E-142 | 1.281367519 | 0.944 | 0.648 | 1.27E-137 | Mφ-FCN1 |
| VCAN | 4.27E-154 | 1.280602199 | 0.855 | 0.281 | 9.82E-150 | Mφ-FCN1 |
| S100A12 | 7.43E-132 | 1.278337642 | 0.702 | 0.154 | 1.71E-127 | Mφ-FCN1 |
| EHD1 | 1.51E-174 | 1.269085868 | 0.858 | 0.376 | 3.48E-170 | Mφ-FCN1 |
| ATP2B1-AS1 | 3.95E-163 | 1.262679517 | 0.863 | 0.402 | 9.09E-159 | Mφ-FCN1 |
| APOBEC3A | 3.05E-71 | 1.257308173 | 0.413 | 0.065 | 7.03E-67 | Mφ-FCN1 |
| CXCL8 | 2.93E-149 | 1.254083366 | 0.971 | 0.714 | 6.75E-145 | Mφ-FCN1 |
| INHBA | 2.38E-65 | 1.223405695 | 0.533 | 0.187 | 5.48E-61 | Mφ-FCN1 |
| GK | 6.20E-170 | 1.21119362 | 0.927 | 0.589 | 1.43E-165 | Mφ-FCN1 |
| TRAF1 | 2.94E-131 | 1.20451033 | 0.687 | 0.211 | 6.77E-127 | Mφ-FCN1 |
| C15orf48 | 1.44E-125 | 1.183659039 | 0.906 | 0.655 | 3.31E-121 | Mφ-FCN1 |
| ATP2B1 | 2.72E-150 | 1.178807403 | 0.879 | 0.541 | 6.27E-146 | Mφ-FCN1 |
| WTAP | 6.03E-148 | 1.144005495 | 0.878 | 0.634 | 1.39E-143 | Mφ-FCN1 |
| OASL | 3.20E-102 | 1.127810227 | 0.578 | 0.165 | 7.38E-98 | Mφ-FCN1 |
| CD44 | 3.11E-197 | 1.125610976 | 0.985 | 0.769 | 7.15E-193 | Mφ-FCN1 |
| SLC25A37 | 2.33E-142 | 1.107489894 | 0.859 | 0.346 | 5.37E-138 | Mφ-FCN1 |
| IVNS1ABP | 4.88E-123 | 1.070781985 | 0.817 | 0.51 | 1.12E-118 | Mφ-FCN1 |
| PPIF | 4.78E-134 | 1.053641591 | 0.931 | 0.723 | 1.10E-129 | Mφ-FCN1 |
| HSPA6 | 8.75E-71 | 1.050404111 | 0.676 | 0.366 | 2.01E-66 | Mφ-FCN1 |
| NAMPT | 5.23E-202 | 1.040863735 | 0.982 | 0.829 | 1.20E-197 | Mφ-FCN1 |
| G0S2 | 1.03E-139 | 1.03077057 | 0.965 | 0.578 | 2.37E-135 | Mφ-FCN1 |
| CXCL1 | 1.86E-15 | 1.027960911 | 0.424 | 0.283 | 4.29E-11 | Mφ-FCN1 |
| DDIT4 | 3.80E-110 | 1.025929857 | 0.855 | 0.565 | 8.75E-106 | Mφ-FCN1 |
| TNFRSF1B | 1.22E-124 | 1.023738832 | 0.909 | 0.709 | 2.80E-120 | Mφ-FCN1 |
| GNG2 | 1.70E-77 | 1.003934103 | 0.555 | 0.219 | 3.91E-73 | Mφ-FCN1 |
| PPBP | 2.02E-96 | 3.396761343 | 0.268 | 0.007 | 4.64E-92 | Mφ-SPP1 |
| CXCL5 | 6.50E-74 | 2.341904412 | 0.54 | 0.105 | 1.50E-69 | Mφ-SPP1 |
| MT2A | 1.26E-27 | 1.631086511 | 0.808 | 0.568 | 2.91E-23 | Mφ-SPP1 |
| ALOX5AP | 2.80E-66 | 1.535867698 | 0.901 | 0.529 | 6.44E-62 | Mφ-SPP1 |
| SPP1 | 9.56E-25 | 1.528690565 | 0.474 | 0.198 | 2.20E-20 | Mφ-SPP1 |
| RETN | 4.37E-78 | 1.423470685 | 0.845 | 0.293 | 1.01E-73 | Mφ-SPP1 |
| IL1R2 | 1.28E-59 | 1.393676589 | 0.437 | 0.082 | 2.96E-55 | Mφ-SPP1 |
| AKAP12 | 5.01E-23 | 1.208333866 | 0.197 | 0.039 | 1.15E-18 | Mφ-SPP1 |
| FOLR3 | 5.89E-122 | 1.187282501 | 0.451 | 0.029 | 1.36E-117 | Mφ-SPP1 |
| THBD | 3.15E-57 | 1.167311509 | 0.756 | 0.286 | 7.26E-53 | Mφ-SPP1 |
| CD163 | 2.76E-56 | 1.123610034 | 0.873 | 0.484 | 6.36E-52 | Mφ-SPP1 |
| MMP19 | 5.30E-43 | 1.080619212 | 0.77 | 0.399 | 1.22E-38 | Mφ-SPP1 |
| MRC1 | 3.65E-38 | 1.074501079 | 0.634 | 0.272 | 8.41E-34 | Mφ-SPP1 |
| ANGPTL4 | 8.17E-58 | 1.072892823 | 0.549 | 0.126 | 1.88E-53 | Mφ-SPP1 |
| FPR2 | 6.85E-45 | 0.948451683 | 0.573 | 0.193 | 1.58E-40 | Mφ-SPP1 |
| SLA | 2.68E-48 | 0.904097679 | 0.784 | 0.374 | 6.17E-44 | Mφ-SPP1 |
| CH25H | 7.28E-50 | 0.884221659 | 0.315 | 0.044 | 1.68E-45 | Mφ-SPP1 |
| FN1 | 0.005627227 | 0.850026706 | 0.122 | 0.074 | 1 | Mφ-SPP1 |
| C1orf162 | 3.40E-36 | 0.804349984 | 0.784 | 0.467 | 7.82E-32 | Mφ-SPP1 |
| RGCC | 5.46E-20 | 0.798407222 | 0.615 | 0.36 | 1.26E-15 | Mφ-SPP1 |
| FKBP5 | 3.43E-51 | 0.781058314 | 0.615 | 0.202 | 7.91E-47 | Mφ-SPP1 |
| NT5E | 7.05E-54 | 0.779304572 | 0.376 | 0.062 | 1.62E-49 | Mφ-SPP1 |
| TSC22D3 | 1.66E-31 | 0.77610513 | 0.765 | 0.442 | 3.82E-27 | Mφ-SPP1 |
| CCL7 | 1.66E-09 | 0.775246412 | 0.136 | 0.042 | 3.83E-05 | Mφ-SPP1 |
| LPL | 1.48E-57 | 0.774628815 | 0.319 | 0.04 | 3.41E-53 | Mφ-SPP1 |
| C5AR1 | 1.32E-23 | 0.773074725 | 0.775 | 0.544 | 3.04E-19 | Mφ-SPP1 |
| DUSP4 | 5.75E-29 | 0.764470854 | 0.653 | 0.314 | 1.32E-24 | Mφ-SPP1 |
| S100A10 | 2.56E-48 | 0.763237552 | 0.972 | 0.877 | 5.90E-44 | Mφ-SPP1 |
| PHLDA1 | 4.25E-24 | 0.760835059 | 0.737 | 0.49 | 9.79E-20 | Mφ-SPP1 |
| CEBPD | 3.60E-20 | 0.759243377 | 0.784 | 0.567 | 8.29E-16 | Mφ-SPP1 |
| MT1E | 2.11E-17 | 0.748138835 | 0.291 | 0.102 | 4.86E-13 | Mφ-SPP1 |
| CTSL | 3.88E-28 | 0.741252836 | 0.958 | 0.837 | 8.93E-24 | Mφ-SPP1 |
| ACSL3 | 5.53E-28 | 0.736441602 | 0.657 | 0.363 | 1.27E-23 | Mφ-SPP1 |
| PLEC | 3.91E-32 | 0.729898221 | 0.671 | 0.337 | 9.00E-28 | Mφ-SPP1 |
| CALM1 | 4.46E-37 | 0.696784603 | 0.944 | 0.861 | 1.03E-32 | Mφ-SPP1 |
| B3GNT5 | 1.82E-29 | 0.696415254 | 0.62 | 0.307 | 4.18E-25 | Mφ-SPP1 |
| TXNIP | 4.87E-25 | 0.685081123 | 0.695 | 0.368 | 1.12E-20 | Mφ-SPP1 |
| SMIM25 | 2.84E-27 | 0.684312854 | 0.789 | 0.507 | 6.55E-23 | Mφ-SPP1 |
| PROCR | 6.19E-25 | 0.671684315 | 0.324 | 0.1 | 1.43E-20 | Mφ-SPP1 |
| CD93 | 5.28E-21 | 0.665898298 | 0.77 | 0.536 | 1.22E-16 | Mφ-SPP1 |
| FBP1 | 1.96E-15 | 0.663812615 | 0.62 | 0.419 | 4.52E-11 | Mφ-SPP1 |
| MIF | 5.09E-27 | 0.6627964 | 0.92 | 0.798 | 1.17E-22 | Mφ-SPP1 |
| MCEMP1 | 2.56E-22 | 0.657407724 | 0.789 | 0.501 | 5.89E-18 | Mφ-SPP1 |
| RNASE1 | 2.56E-18 | 0.6558903 | 0.493 | 0.232 | 5.91E-14 | Mφ-SPP1 |
| TUBA1C | 8.69E-21 | 0.635911954 | 0.704 | 0.493 | 2.00E-16 | Mφ-SPP1 |
| CA12 | 3.39E-63 | 0.635550072 | 0.192 | 0.007 | 7.80E-59 | Mφ-SPP1 |
| PLIN2 | 2.14E-28 | 0.624934487 | 0.934 | 0.805 | 4.93E-24 | Mφ-SPP1 |
| LRRFIP1 | 2.57E-29 | 0.623603378 | 0.864 | 0.65 | 5.92E-25 | Mφ-SPP1 |
| S100A6 | 1.18E-34 | 0.609569023 | 0.995 | 0.919 | 2.72E-30 | Mφ-SPP1 |
| ANXA1 | 1.84E-19 | 0.608848373 | 0.84 | 0.697 | 4.24E-15 | Mφ-SPP1 |

### Table S4 Gene signature

| Genes | Signature | Dataset |
| --- | --- | --- |
| ZWINT | proliferation | |
| E2F1 | proliferation | |
| FEN1 | proliferation | |
| FOXM1 | proliferation | |
| H2AFZ | proliferation | |
| HMGB2 | proliferation | |
| MCM2 | proliferation | |
| MCM3 | proliferation | |
| MCM4 | proliferation | |
| MCM5 | proliferation | |
| MCM6 | proliferation | |
| MKI67 | proliferation | |
| MYBL2 | proliferation | |
| PCNA | proliferation | |
| PLK1 | proliferation | |
| CCND1 | proliferation | |
| AURKA | proliferation | |
| BUB1 | proliferation | |
| TOP2A | proliferation | |
| TYMS | proliferation | |
| DEK | proliferation | |
| CCNB1 | proliferation | |
| CCNE1 | proliferation | |
| VEGFA | Hypoxia | Buffa |
| SLC2A1 | Hypoxia | Buffa |
| PGAM1 | Hypoxia | Buffa |
| ENO1 | Hypoxia | Buffa |
| LDHA | Hypoxia | Buffa |
| TPI1 | Hypoxia | Buffa |
| P4HA1 | Hypoxia | Buffa |
| MRPS17 | Hypoxia | Buffa |
| CDKN3 | Hypoxia | Buffa |
| ADM | Hypoxia | Buffa |
| NDRG1 | Hypoxia | Buffa |
| TUBB6 | Hypoxia | Buffa |
| ALDOA | Hypoxia | Buffa |
| MIF | Hypoxia | Buffa |
| ACOT7 | Hypoxia | Buffa |
| MCTS1 | Hypoxia | Buffa |
| PSRC1 | Hypoxia | Buffa |
| PSMA7 | Hypoxia | Buffa |
| ANLN | Hypoxia | Buffa |
| TUBA1B | Hypoxia | Buffa |
| MAD2L2 | Hypoxia | Buffa |
| GPI | Hypoxia | Buffa |
| TUBA1C | Hypoxia | Buffa |
| MAP7D1 | Hypoxia | Buffa |
| DDIT4 | Hypoxia | Buffa |
| BNIP3 | Hypoxia | Buffa |
| C20orf20 | Hypoxia | Buffa |
| HIG2 | Hypoxia | Buffa |
| GAPDH | Hypoxia | Buffa |
| MRPL13 | Hypoxia | Buffa |
| CHCHD2 | Hypoxia | Buffa |
| YKT6 | Hypoxia | Buffa |
| NP | Hypoxia | Buffa |
| CORO1C | Hypoxia | Buffa |
| SEC61G | Hypoxia | Buffa |
| ANKRD37 | Hypoxia | Buffa |
| ESRP1 | Hypoxia | Buffa |
| PFKP | Hypoxia | Buffa |
| SHCBP1 | Hypoxia | Buffa |
| CTSL2 | Hypoxia | Buffa |
| KIF20A | Hypoxia | Buffa |
| SLC25A32 | Hypoxia | Buffa |
| UTP11L | Hypoxia | Buffa |
| SLC16A1 | Hypoxia | Buffa |
| MRPL15 | Hypoxia | Buffa |
| KIF4A | Hypoxia | Buffa |
| LRRC42 | Hypoxia | Buffa |
| PGK1 | Hypoxia | Buffa |
| HK2 | Hypoxia | Buffa |
| AK3L1 | Hypoxia | Buffa |
| CA9 | Hypoxia | Buffa |
| ALDOA | Hypoxia | West |
| ANGPTL4 | Hypoxia | West |
| ANLN | Hypoxia | West |
| BNC1 | Hypoxia | West |
| C20orf20 | Hypoxia | West |
| CA9 | Hypoxia | West |
| CDKN3 | Hypoxia | West |
| COL4A6 | Hypoxia | West |
| DCBLD1 | Hypoxia | West |
| ENO1 | Hypoxia | West |
| FAM83B | Hypoxia | West |
| FOSL1 | Hypoxia | West |
| GNAI1 | Hypoxia | West |
| HIG2 | Hypoxia | West |
| KCTD11 | Hypoxia | West |
| KRT17 | Hypoxia | West |
| LDHA | Hypoxia | West |
| MPRS17 | Hypoxia | West |
| P4HA1 | Hypoxia | West |
| PGAM1 | Hypoxia | West |
| PGK1 | Hypoxia | West |
| SDC1 | Hypoxia | West |
| SLC16A1 | Hypoxia | West |
| SLC2A1 | Hypoxia | West |
| TPI1 | Hypoxia | West |
| VEGFA | Hypoxia | West |
| MTX1 | Hypoxia | Winter |
| ADORA2B | Hypoxia | Winter |
| AK3 | Hypoxia | Winter |
| ALDOA | Hypoxia | Winter |
| ANGPTL4 | Hypoxia | Winter |
| C20orf20 | Hypoxia | Winter |
| MRPS17 | Hypoxia | Winter |
| PGF | Hypoxia | Winter |
| PGK1 | Hypoxia | Winter |
| AFARP1 | Hypoxia | Winter |
| ANLN | Hypoxia | Winter |
| B4GALT2 | Hypoxia | Winter |
| BCAR1 | Hypoxia | Winter |
| BMS1L | Hypoxia | Winter |
| BNIP3 | Hypoxia | Winter |
| HOMER1 | Hypoxia | Winter |
| HSPC163 | Hypoxia | Winter |
| IMP-2 | Hypoxia | Winter |
| KIAA1393 | Hypoxia | Winter |
| LDHA | Hypoxia | Winter |
| LDLR | Hypoxia | Winter |
| MGC2654 | Hypoxia | Winter |
| MNAT1 | Hypoxia | Winter |
| NDRG1 | Hypoxia | Winter |
| NME1 | Hypoxia | Winter |
| P4HA1 | Hypoxia | Winter |
| PFKFB4 | Hypoxia | Winter |
| PGAM1 | Hypoxia | Winter |
| PVR | Hypoxia | Winter |
| SLC16A1 | Hypoxia | Winter |
| SLC2A1 | Hypoxia | Winter |
| TEAD4 | Hypoxia | Winter |
| TPBG | Hypoxia | Winter |
| TPI1 | Hypoxia | Winter |
| TUBB2 | Hypoxia | Winter |
| VEGF | Hypoxia | Winter |
| VEZT | Hypoxia | Winter |
| AD-003 | Hypoxia | Winter |
| ANKRD9 | Hypoxia | Winter |
| C14orf156 | Hypoxia | Winter |
| C15orf25 | Hypoxia | Winter |
| CA12 | Hypoxia | Winter |
| CA9 | Hypoxia | Winter |
| CDCA4 | Hypoxia | Winter |
| COL4A5 | Hypoxia | Winter |
| CORO1C | Hypoxia | Winter |
| CTEN | Hypoxia | Winter |
| DKFZP564D166 | Hypoxia | Winter |
| DPM2 | Hypoxia | Winter |
| EIF2S1 | Hypoxia | Winter |
| GAPD | Hypoxia | Winter |
| GMFB | Hypoxia | Winter |
| GSS | Hypoxia | Winter |
| HES2 | Hypoxia | Winter |
| HIG2 | Hypoxia | Winter |
| IL8 | Hypoxia | Winter |
| KCTD11 | Hypoxia | Winter |
| KRT17 | Hypoxia | Winter |
| Kua | Hypoxia | Winter |
| LOC149464 | Hypoxia | Winter |
| LOC56901 | Hypoxia | Winter |
| LRP2BP | Hypoxia | Winter |
| MGC14560 | Hypoxia | Winter |
| MGC17624 | Hypoxia | Winter |
| MGC2408 | Hypoxia | Winter |
| MIF | Hypoxia | Winter |
| MRPL14 | Hypoxia | Winter |
| NUDT15 | Hypoxia | Winter |
| PAWR | Hypoxia | Winter |
| PDZK11 | Hypoxia | Winter |
| PLAU | Hypoxia | Winter |
| PLEKHG3 | Hypoxia | Winter |
| PPARD | Hypoxia | Winter |
| PPP2CZ | Hypoxia | Winter |
| PPP4R1 | Hypoxia | Winter |
| PSMA7 | Hypoxia | Winter |
| PSMB7 | Hypoxia | Winter |
| PSMD2 | Hypoxia | Winter |
| PTGFRN | Hypoxia | Winter |
| PYGL | Hypoxia | Winter |
| RAN | Hypoxia | Winter |
| RNF24 | Hypoxia | Winter |
| RNPS1 | Hypoxia | Winter |
| RUVBL2 | Hypoxia | Winter |
| S100A10 | Hypoxia | Winter |
| S100A3 | Hypoxia | Winter |
| SIP1 | Hypoxia | Winter |
| SLC6A10 | Hypoxia | Winter |
| SLC6A8 | Hypoxia | Winter |
| SLCO1B3 | Hypoxia | Winter |
| SMILE | Hypoxia | Winter |
| SNX24 | Hypoxia | Winter |
| SPTB | Hypoxia | Winter |
| TFAP2C | Hypoxia | Winter |
| TIMM23 | Hypoxia | Winter |
| TMEM30B | Hypoxia | Winter |
| TPD52L2 | Hypoxia | Winter |
| VAPB | Hypoxia | Winter |
| XPO5 | Hypoxia | Winter |
| TIGIT | Anti_inflammatory | |
| IDO1 | Anti_inflammatory | |
| LGALS3 | Anti_inflammatory | |
| PDCD1 | Anti_inflammatory | |
| FOXP3 | Anti_inflammatory | |
| ENTPD1 | Anti_inflammatory | |
| CD274 | Anti_inflammatory | |
| CSF2 | Anti_inflammatory | |
| CTLA4 | Anti_inflammatory | |
| CXCL12 | Anti_inflammatory | |
| CXCL5 | Anti_inflammatory | |
| IL8 | Anti_inflammatory | |
| MIF | Anti_inflammatory | |
| PTGS2 | Anti_inflammatory | |
| VEGFA | Anti_inflammatory | |
| IL1A | Pro_inflammatory | |
| IL1B | Pro_inflammatory | |
| TNF | Pro_inflammatory | |
| IFNG | Pro_inflammatory | |
| TBX21 | Pro_inflammatory | |
| CCL3 | Pro_inflammatory | |
| CCL4 | Pro_inflammatory | |
| PRF1 | Pro_inflammatory | |
| GZMA | Pro_inflammatory | |
| GZMB | Pro_inflammatory | |
| GZMK | Pro_inflammatory | |
| GZMH | Pro_inflammatory | |
| CD8A | Pro_inflammatory | |
| FASLG | Pro_inflammatory | |
| CCL2 | Pro_inflammatory | |
| CCL20 | Pro_inflammatory | |
| IL2 | Pro_inflammatory | |
| IL6 | Pro_inflammatory | |
| IL12a | Pro_inflammatory | |
| IL17a | Pro_inflammatory | |
| IL23a | Pro_inflammatory | |
| PTGS2 | Pro_inflammatory | |
| TLR4 | Pro_inflammatory | |
| TNF | Pro_inflammatory | |
| IL12 | M1_Macrophage_Polarization | |
| IL23 | M1_Macrophage_Polarization | |
| IL12 | M1_Macrophage_Polarization | |
| TNF | M1_Macrophage_Polarization | |
| IL6 | M1_Macrophage_Polarization | |
| CD86 | M1_Macrophage_Polarization | |
| MHCII | M1_Macrophage_Polarization | |
| IL1B | M1_Macrophage_Polarization | |
| MARCO | M1_Macrophage_Polarization | |
| iNOS | M1_Macrophage_Polarization | |
| IL12 | M1_Macrophage_Polarization | |
| CD64 | M1_Macrophage_Polarization | |
| CD80 | M1_Macrophage_Polarization | |
| CXCR10 | M1_Macrophage_Polarization | |
| IL23 | M1_Macrophage_Polarization | |
| CXCL9 | M1_Macrophage_Polarization | |
| CXCL10 | M1_Macrophage_Polarization | |
| CXCL11 | M1_Macrophage_Polarization | |
| CD86 | M1_Macrophage_Polarization | |
| IL1A | M1_Macrophage_Polarization | |
| IL1B | M1_Macrophage_Polarization | |
| IL6 | M1_Macrophage_Polarization | |
| TNFa | M1_Macrophage_Polarization | |
| MHCII | M1_Macrophage_Polarization | |
| CCL5 | M1_Macrophage_Polarization | |
| IRF5 | M1_Macrophage_Polarization | |
| IRF1 | M1_Macrophage_Polarization | |
| CD40 | M1_Macrophage_Polarization | |
| IDO1 | M1_Macrophage_Polarization | |
| KYNU | M1_Macrophage_Polarization | |
| CCR7 | M1_Macrophage_Polarization | |
| CD45 | M1_Macrophage_Polarization | |
| CD68 | M1_Macrophage_Polarization | |
| CD115 | M1_Macrophage_Polarization | |
| HLA-DR | M1_Macrophage_Polarization | |
| CD205 | M1_Macrophage_Polarization | |
| CD14 | M1_Macrophage_Polarization | |
| ARG1 | M2_Macrophage_Polarization | |
| ARG2 | M2 Macrophage Polarization | |
| IL10 | M2 Macrophage Polarization | |
| CD32 | M2 Macrophage Polarization | |
| CD163 | M2 Macrophage Polarization | |
| CD23 | M2 Macrophage Polarization | |
| CD200R1 | M2 Macrophage Polarization | |
| PD-L2 | M2 Macrophage Polarization | |
| PDL1 | M2 Macrophage Polarization | |
| MARCO | M2 Macrophage Polarization | |
| CSF1R | M2 Macrophage Polarization | |
| CD206 | M2 Macrophage Polarization | |
| IL1RN | M2 Macrophage Polarization | |
| IL1R2 | M2 Macrophage Polarization | |
| IL4R | M2 Macrophage Polarization | |
| CCL4 | M2 Macrophage Polarization | |
| CCL13 | M2 Macrophage Polarization | |
| CCL20 | M2 Macrophage Polarization | |
| CCL17 | M2 Macrophage Polarization | |
| CCL18 | M2 Macrophage Polarization | |
| CCL22 | M2 Macrophage Polarization | |
| CCL24 | M2 Macrophage Polarization | |
| LYVE1 | M2 Macrophage Polarization | |
| VEGFA | M2 Macrophage Polarization | |
| VEGFB | M2 Macrophage Polarization | |
| VEGFC | M2 Macrophage Polarization | |
| VEGFD | M2 Macrophage Polarization | |
| EGF | M2 Macrophage Polarization | |
| CTSA | M2 Macrophage Polarization | |
| CTSB | M2 Macrophage Polarization | |
| CSTC | M2 Macrophage Polarization | |
| CTSD | M2 Macrophage Polarization | |
| TGFB1 | M2 Macrophage Polarization | |
| TGFB2 | M2 Macrophage Polarization | |
| TGFB3 | M2 Macrophage Polarization | |
| MMP14 | M2 Macrophage Polarization | |
| MMP19 | M2 Macrophage Polarization | |
| MMP9 | M2 Macrophage Polarization | |
| CLEC7A | M2 Macrophage Polarization | |
| WNT7B | M2 Macrophage Polarization | |
| FASL | M2 Macrophage Polarization | |
| TNFSF12 | M2 Macrophage Polarization | |
| TNFSF8 | M2 Macrophage Polarization | |
| CD276 | M2 Macrophage Polarization | |
| VTCN1 | M2 Macrophage Polarization | |
| MSR1 | M2 Macrophage Polarization | |
| FN1 | M2 Macrophage Polarization | |
| IRF4 | M2 Macrophage Polarization | |
| CD45 | M2 Macrophage Polarization | |
| CD68 | M2 Macrophage Polarization | |
| CD115 | M2 Macrophage Polarization | |
| HLA-DR | M2 Macrophage Polarization | |
| CD205 | M2 Macrophage Polarization | |
| CD14 | M2 Macrophage Polarization | |
| HAVCR2 | CD8_Exhausted | |
| CXCL13 | CD8_Exhausted | |
| CCL3 | CD8_Exhausted | |
| SIRPG | CD8_Exhausted | |
| IFNG | CD8_Exhausted | |
| TIGIT | CD8_Exhausted | |
| GZMB | CD8_Exhausted | |
| PDCD1 | CD8_Exhausted | |
| PARK7 | CD8_Exhausted | |
| TNFRSF9 | CD8_Exhausted | |
| ACP5 | CD8_Exhausted | |
| CTLA4 | CD8_Exhausted | |
| RBPJ | CD8_Exhausted | |
| MIR155 | CD8_Exhausted | |
| CXCR6 | CD8_Exhausted | |
| CD27 | CD8_Exhausted | |
| FKBP1A | CD8_Exhausted | |
| BST2 | CD8_Exhausted | |
| TPI1 | CD8_Exhausted | |
| MIR155HG | CD8_Exhausted | |
| PTTG1 | CD8_Exhausted | |
| CD63 | CD8_Exhausted | |
| SAMSN1 | CD8_Exhausted | |
| RGS1 | CD8_Exhausted | |
| CD27-AS1 | CD8_Exhausted | |
| ITGAE | CD8_Exhausted | |
| MIR4632 | CD8_Exhausted | |
| HLA-DRA | CD8_Exhausted | |
| IGFLR1 | CD8_Exhausted | |
| KRT86 | CD8_Exhausted | |
| ENTPD1 | CD8_Exhausted | |
| DUSP4 | CD8_Exhausted | |
| SIT1 | CD8_Exhausted | |
| TOX | CD8_Exhausted | |
| PHLDA1 | CD8_Exhausted | |
| CCND2 | CD8_Exhausted | |
| GPR25 | CD8_Exhausted | |
| LAYN | CD8_Exhausted | |
| PRDX5 | CD8_Exhausted | |
| SARDH | CD8_Exhausted | |
| FASLG | CD8_Exhausted | |
| MIR3917 | CD8_Exhausted | |
| ANXA5 | CD8_Exhausted | |
| CTSD | CD8_Exhausted | |
| PDIA6 | CD8_Exhausted | |
| RANBP1 | CD8_Exhausted | |
| FKBP1A-SDCBP2 | CD8_Exhausted | |
| COTL1 | CD8_Exhausted | |
| TNFRSF1B | CD8_Exhausted | |
| IDH2 | CD8_Exhausted | |
| CD38 | CD8_Exhausted | |
| CD82 | CD8_Exhausted | |
| LAG3 | CD8_Exhausted | |
| MIR497HG | CD8_Exhausted | |
| APOBEC3C | CD8_Exhausted | |
| ITM2A | CD8_Exhausted | |
| COX5A | CD8_Exhausted | |
| IFI35 | CD8_Exhausted | |
| NDFIP2 | CD8_Exhausted | |
| TNFRSF18 | CD8_Exhausted | |
| KRT81 | CD8_Exhausted | |
| DNPH1 | CD8_Exhausted | |
| RGS2 | CD8_Exhausted | |
| HMGN1 | CD8_Exhausted | |
| DYNLL1 | CD8_Exhausted | |
| SNRPB | CD8_Exhausted | |
| STRA13 | CD8_Exhausted | |
| SYNGR2 | CD8_Exhausted | |
| RAB27A | CD8_Exhausted | |
| PSMC3 | CD8_Exhausted | |
| GALM | CD8_Exhausted | |
| FABP5 | CD8_Exhausted | |
| UBE2L6 | CD8_Exhausted | |
| MYO7A | CD8_Exhausted | |
| PRDX3 | CD8_Exhausted | |
| DDIT4 | CD8_Exhausted | |
| STMN1 | CD8_Exhausted | |
| CDK2AP2 | CD8_Exhausted | |
| VCAM1 | CD8_Exhausted | |
| SNAP47 | CD8_Exhausted | |
| PSMB3 | CD8_Exhausted | |
| ISG15 | CD8_Exhausted | |
| HLA-DRB5 | CD8_Exhausted | |
| CKS2 | CD8_Exhausted | |
| TNIP3 | CD8_Exhausted | |
| CD7 | CD8_Exhausted | |
| PSMD4 | CD8_Exhausted | |
| ATP6V1C2 | CD8_Exhausted | |
| PSMD8 | CD8_Exhausted | |
| HLA-DRB6 | CD8_Exhausted | |
| PRF1 | Cytotoxicity | |
| IFNG | Cytotoxicity | |
| GNLY | Cytotoxicity | |
| NKG7 | Cytotoxicity | |
| GZMB | Cytotoxicity | |
| GZMA | Cytotoxicity | |
| GZMH | Cytotoxicity | |
| KLRK1 | Cytotoxicity | |
| KLRB1 | Cytotoxicity | |
| KLRD1 | Cytotoxicity | |
| CTSW | Cytotoxicity | |
| CST7 | Cytotoxicity |  |

## Supplemental Figures


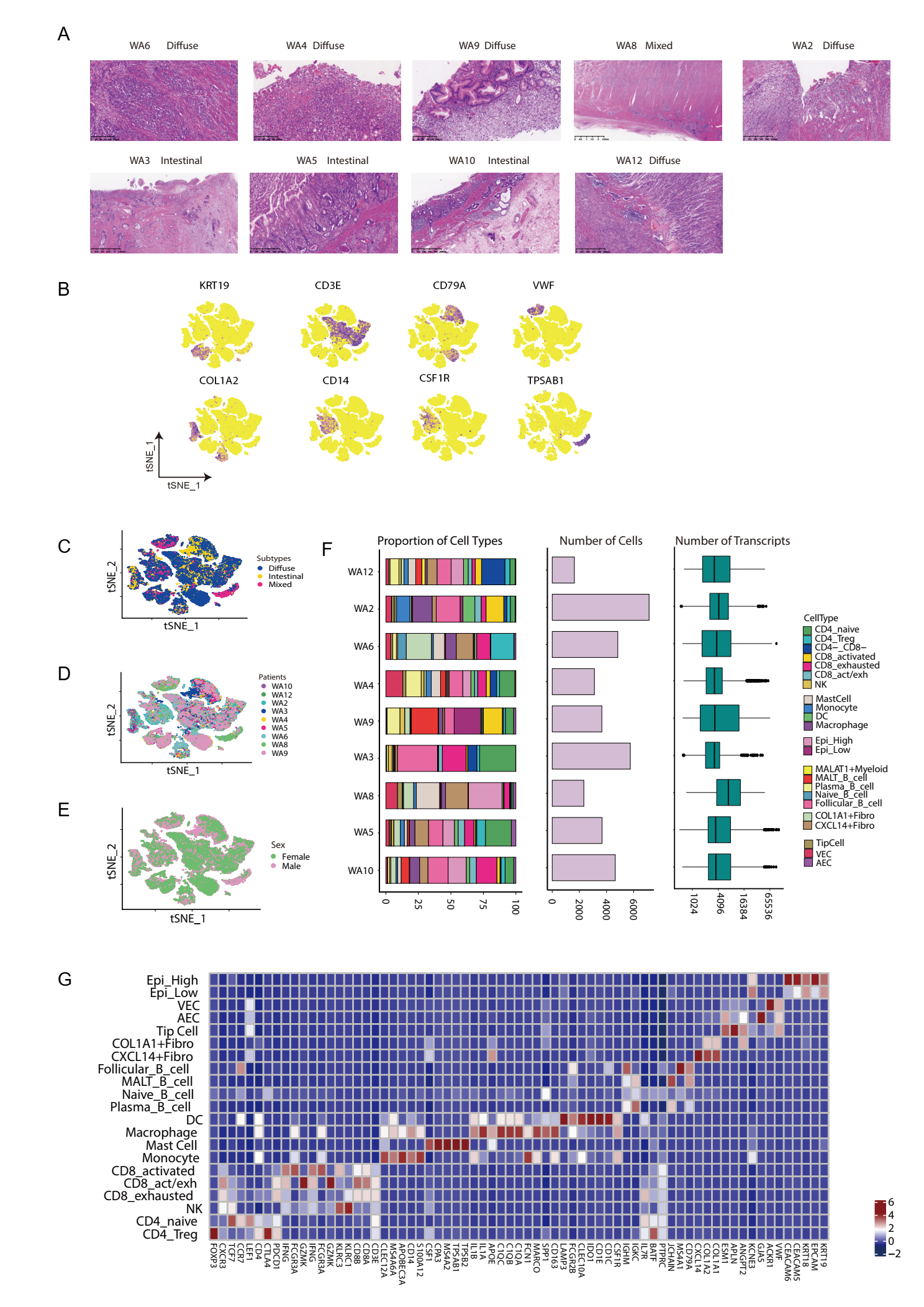


### **Figure S1. Identification of cell subsets and the corresponding marker genes.**

A. Representative images of hematoxylin and eosin staining of FFPE tissue from 9 GAC patients.

B. The classic marker genes that are used for lineage annotation of the six cell meta-clusters (Epithelial cell: *KRT19*; Fibroblast: *COL1A2*; T cell: *CD3E*; Myeloid cell: *CD14, CSF1R* and *TPSAB1*; B cell: *CD79A*; Endothelial cell: *VWF*).

C-E. t-SNE plots that show the distribution of cells were generated according to the (C) histopathological classification, (D) individual, (E) sex, respectively.

F. Cell sub-lineage compositions of GAC patients inferred by scRNA-seq data. Left histogram shows both inter- and intra-patient variations in the proportion of diverse cell sub-lineages (x-axis: proportion of cell subcluster; y-axis: patient ID. Cell subclusters of each sample are color-coded according to the annotation bar on the right side). Middle histogram illustrates the number of cells in each sample (x-axis: the count of cells). Right histogram presents the distribution of transcripts across samples (x-axis: the log_2_ value of transcripts; the median value of transcript number in each sample is indicated by the line that divides the box into two parts).

G. Heatmap that shows the expression of marker genes across cell subclusters. Left: the annotated cell subclusters; bottom: marker genes of different cell subclusters.

###
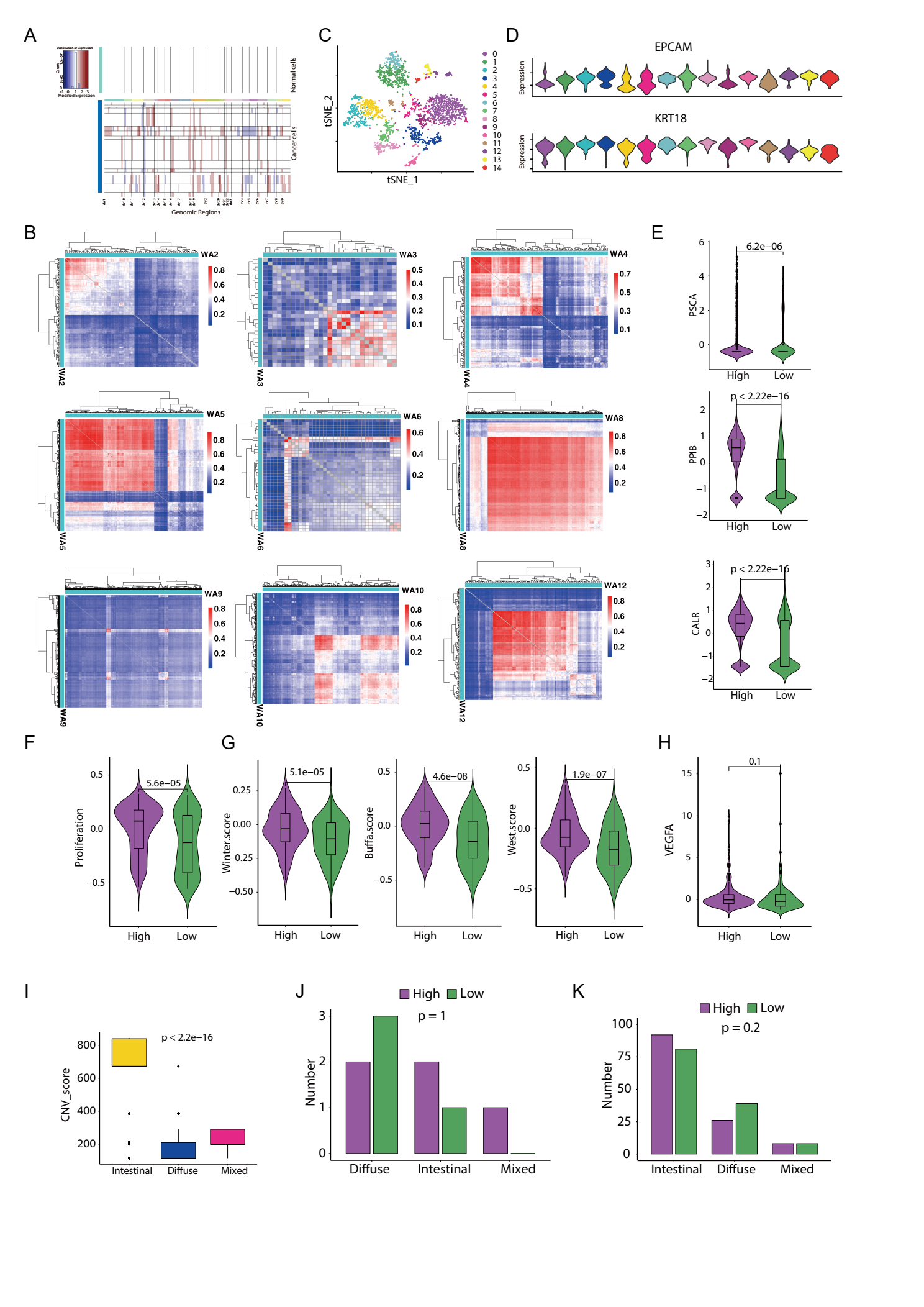
**Figure S2. Similarities and characteristics of ITH-H and ITH-L samples.**

**A.** The distribution of copy number variants across color-coded genomic regions in normal (fibroblast cells and endothelia cells) and cancer cell groups. Cancer cells are referred to as abnormal epithelial cells identified within tumor samples (see **Supplemental Methods**). The modified expression level of genes was inferred from each single cell, and the distribution is shown in the histogram at the left-hand side; x-axis: different genomic regions; blue color: copy loss; red color: copy gain; white color: copy number normal.

**B.** The variation in gene expression pattern among and within patients reveals dramatically high intra- and inter-patient heterogeneity.

**C.** t-SNE plot showing 15 epithelial cell clusters.

**D.** The expression level of two classic GAC biomarkers (EPCAM and KRT18) in each epithelial cell cluster as shown in (B).

**E.** Three genes (PSCA, PPIB, CALR) were identified to be highly expressed in ITH-H samples. These three violin plots show the expression level of the three genes in each ITH group of our cohort.

**F.** The violin plot showing the proliferation score of ITH-H and ITH-L sample groups in TCGA-STAD cohort.

**G.** Three violin plots that illustrate the degree of hypoxia in ITH-H and ITH-L groups in TCGA-STAD cohort based on different quantitation methods.

**H.** Comparison of VEGFA expression between ITH-H and ITH-L groups in the TCGA-STAD cohort.

**I.** Boxplot showing a significantly higher CNV score in the intestinal group comparing to the other histotypes.

**J.** Histogram that shows the number of samples in each group in terms of histotype and ITH degree classification of the samples in our study. There was no correlation between ITH degree classification and histotype.

**K.** Histogram that shows the number of samples in each group in terms of histotype and ITH degree classification of the samples in TCGA cohort.


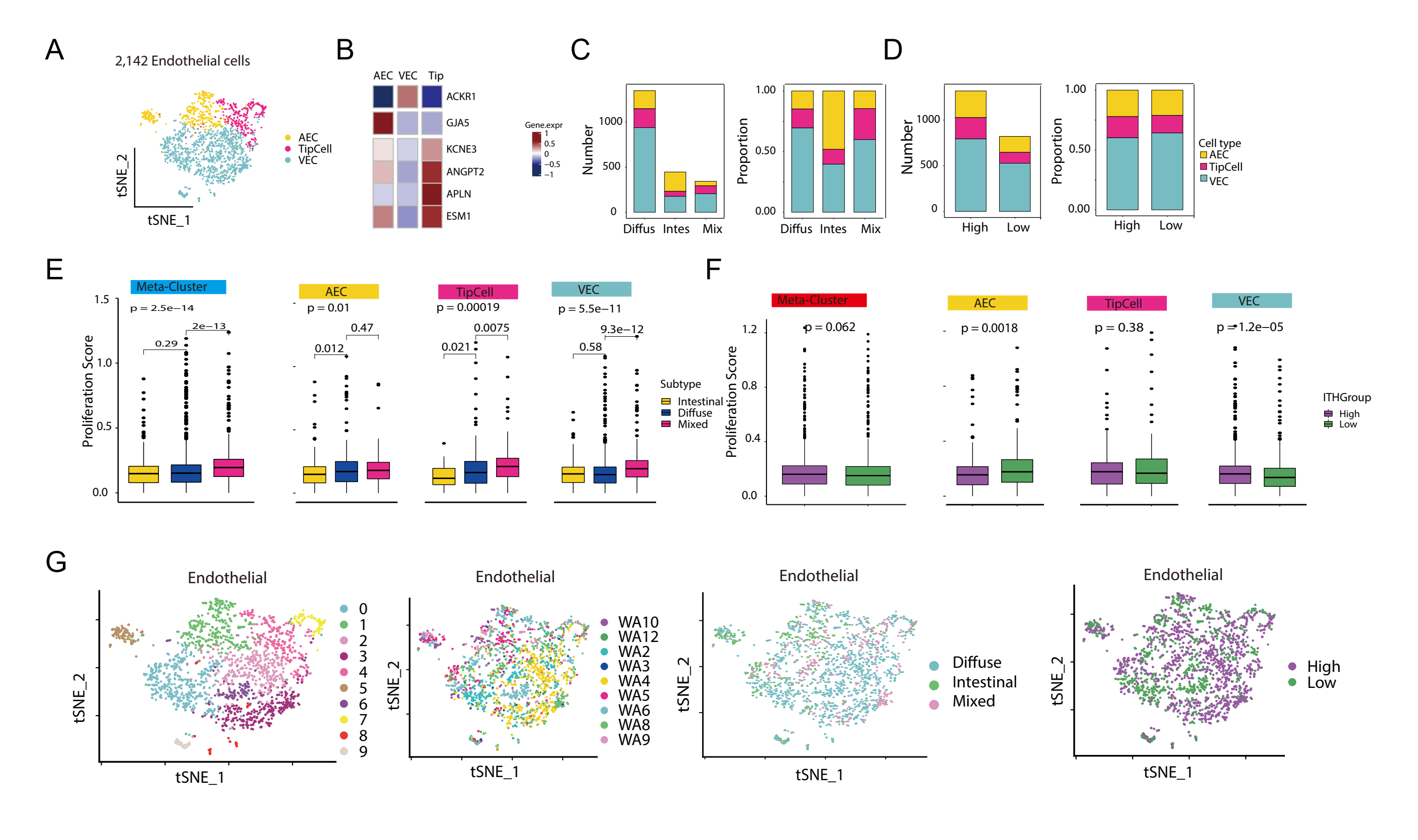


### **Figure S3. Overall distribution and proliferation ability of endothelial cells.**

A. t-SNE projection of 2,142 endothelial cells reveals the existence of three subclusters (color-coded according to the annotation bar).

B. Marker genes that were identified to be differently expressed among three subclusters shown in (**A**) (AEC: GJA5; VEC: ACKR1; Tip cell: KCNE3, ANGPT2, APLN, ESM1).

C. The distribution of three endothelial cell lineages in tumors with different histotypes (left: cell count; right: cell proportion).

D. The distribution of three endothelial cell lineages in tumors with different ITH degrees (left: cell count; right: cell proportion).

E. The proliferation score in the meta-cluster and each subcluster of endothelial cells within tumors with different histotypes.

F. The proliferation score in the meta-cluster and each subcluster of endothelial cells within tumors grouped by ITH degrees.

G. t-SNE plots of endothelial cells from our cohort that are color-coded by cell sub-lineages, patient, histopathological types, ITH degree classifications, respectively.

AEC, artery endothelial cell; VEC, venous endothelial cell.


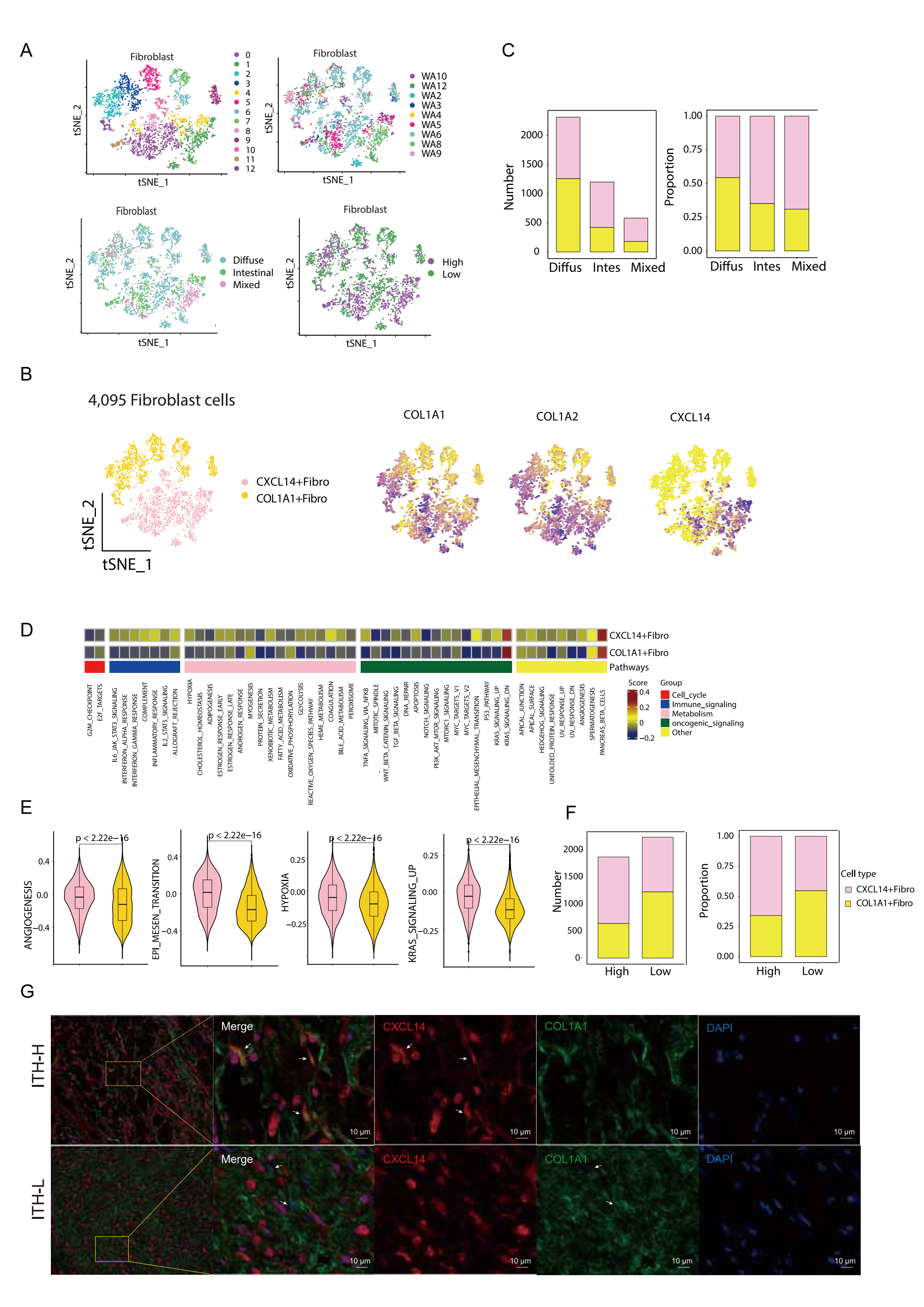


### **Figure S4.** ITH-H tumors enriched for cancer-associated fibroblast recruitment.

A. t-SNE plots of fibroblast cells from our cohort that are color-coded by cell sub-lineages, patient information, histopathological type, ITH degree classification, respectively.

**B.** t-SNE projection of 4,095 fibroblast cells (left). Plots at right side reveal the distribution of fibroblast cells in terms of the expression level of *COL1A1*, *COL1A2*, *CXCL14*, respectively. Two cell subclusters were determined and noted as *COL1A1+*fibroblasts and *CXCL14+* fibroblasts.

C. The distribution of two fibroblast sub-lineages in tumors grouped by histotypes.

**D.** Heatmap that shows the scores of Hallmark pathways in *CXCL14-* fibroblasts and *CXCL14+* fibroblasts.

**E.** Comparisons of the pathway activity related to angiogenesis, EMT transition, hypoxia, upregulation of the expression of *KRAS* gene, between *CXCL14-* fibroblast cells and *CXCL14+* fibroblast cells. Cell type is annotated as shown in the bar on the right side.

**F.** The distribution of two fibroblast sub-lineages in tumors grouped by ITH degree.

**G.** The mIF images of CXCL14 and COL1A1 in GAC tissues from the representative patients with ITH-H (upper) and ITH-L (lower). Scale bar = 10 μm. White arrows, *CXCL14+* fibroblast cells (upper) / *CXCL14-* fibroblast cells (lower).


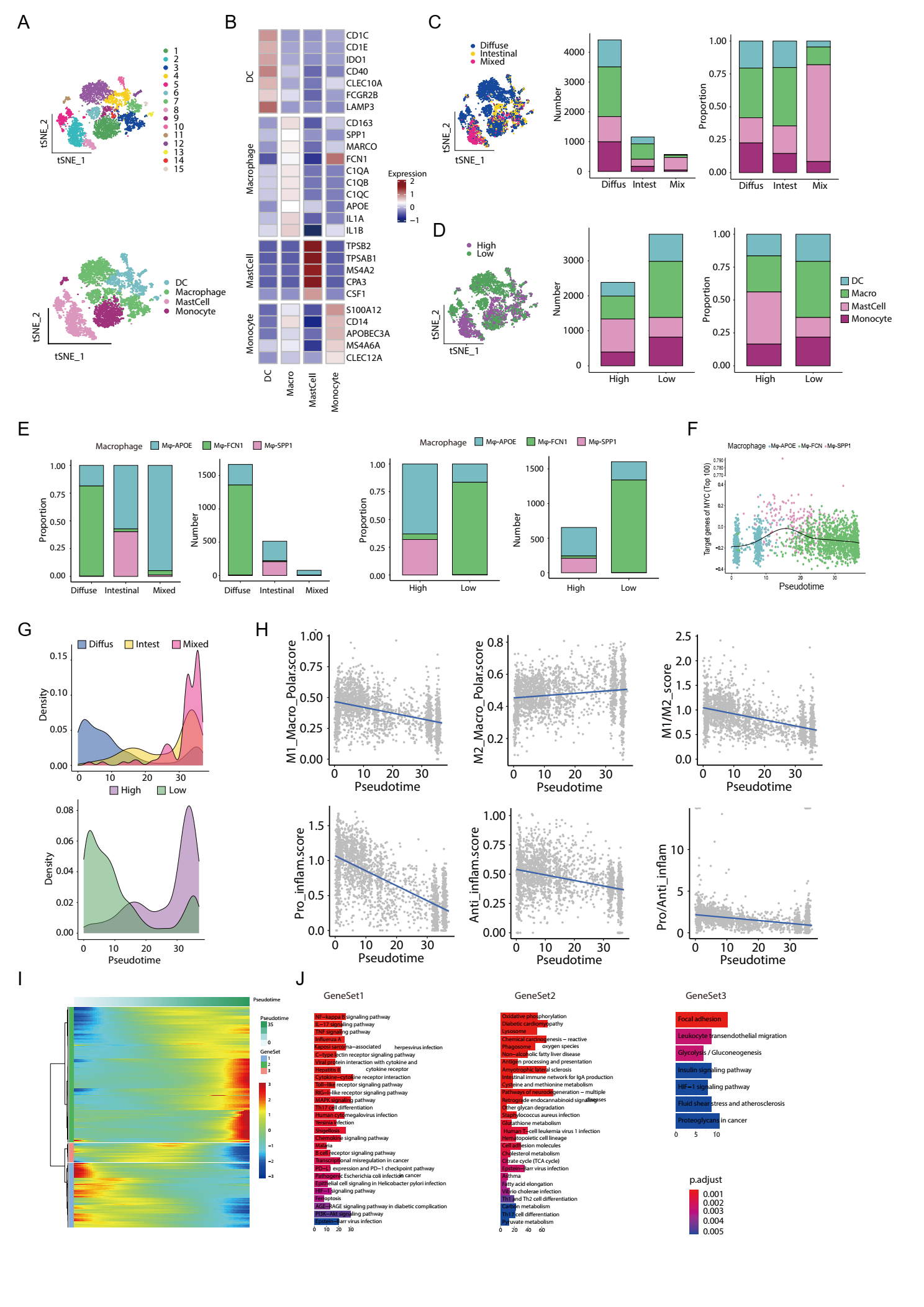


### **Figure S5.** Comprehensive analysis in myeloid cells uncovered a novel intermediate macrophage sub-lineage and M2 polarization in ITH-H tumors.

A. t-SNE plot of 6,289 myeloid cells color-coded by 15 identified clusters (top) and by annotated cell types including DC, macrophage, mast cell and monocyte (bottom).

B. The heatmap that reveals the expression level of several marker genes for four cell lineages shown in (**A**).

C. t-SNE plot of myeloid cells colored by histotype of samples (left); The distribution of four myeloid cell lineages in tumors grouped by histotypes (two histograms on the right side).

D. t-SNE plot of myeloid cells colored by the ITH degree of samples (left); The distribution of four myeloid cell lineages in tumors grouped by ITH degree (two histograms on the right side).

E. The distribution (in terms of proportion and number) of four color-coded macrophage cell sub-lineages in tumors that are grouped by histotypes (left) and by ITH degree classifications (right).

F. The relative expression (estimated by GSVA) of top 100 target genes of MYC (estimated by pySCENIC) along the pseudotime.

G. Density plots that show the change in the distribution of cells grouped by either histotypes (top) or ITH degrees (bottom) of tumor samples with increasing pseudotime.

H. Top: with increasing pseudotime, the polarization score of M1- and M2- macrophage and the ratio of two scores change accordingly. Bottom: with increasing pseudotime, the pro-inflammation and anti-inflammation score and the ratio of two scores, dramatically decrease.

I. Heatmap that shows the change in expression level of 3 sets of genes that are related to macrophage cellular development, with increasing pseudotime. Three gene sets are color-coded as shown.

J. Enrichment of different KEGG pathways using gene set of each cluster is shown in GeneSet 1, GeneSet 2, and GeneSet 3, respectively.


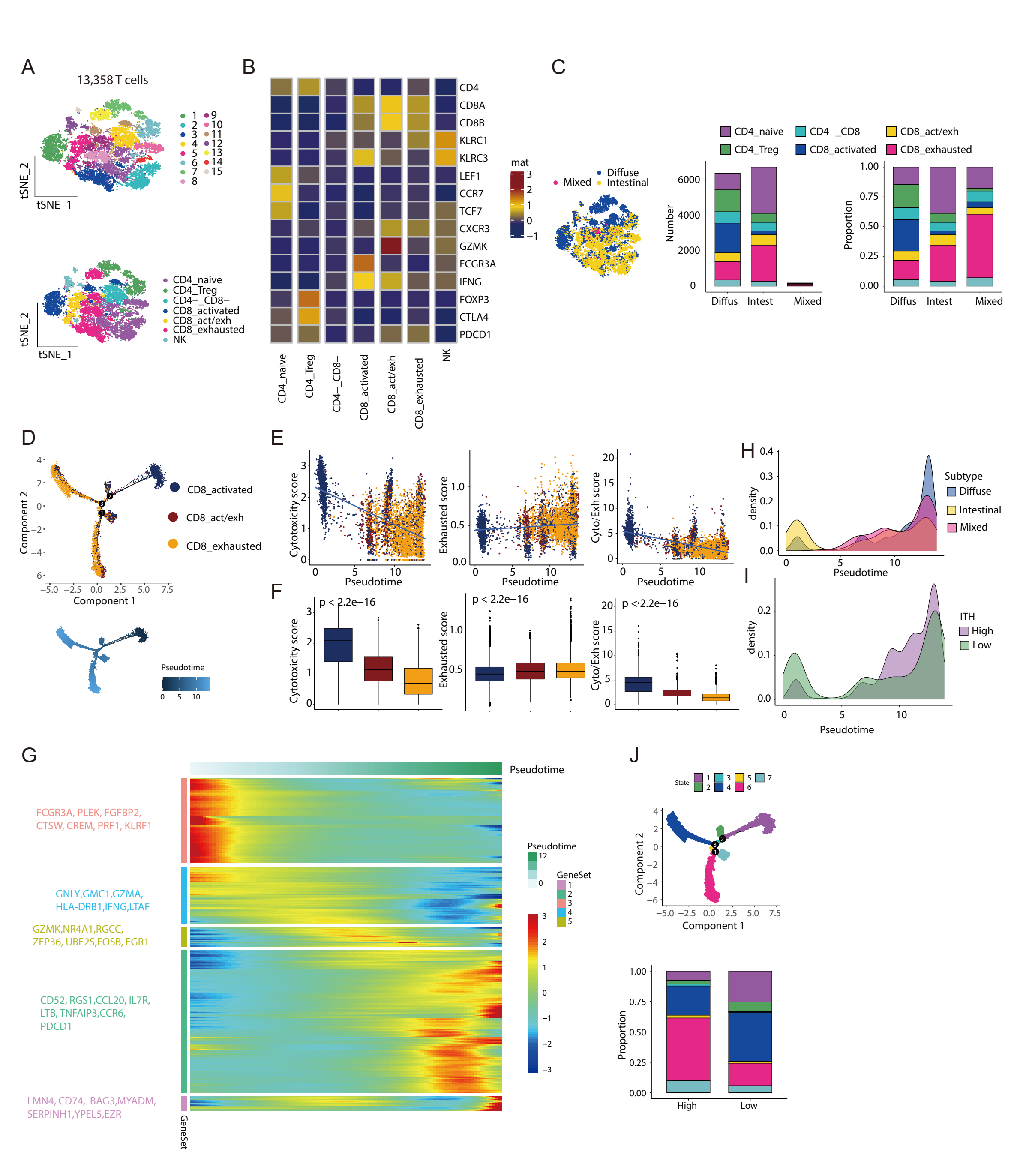


### Figure S6. T cells within ITH-H tumors exhibited exhaustion.

A. t-SNE plot of 13,358 T cells color-coded by fifteen identified clusters (top) and seven cell subsets (bottom).

B. Heatmap of selected T cell marker genes in each cell cluster, as shown in (A).

C. Left: t-SNE plot that shows the distribution of T cells with different histotypes. Right histograms: The count and proportion of different types of T cells in each histotype.

D. Unsupervised transcriptional trajectory of T cells from Monocle (version 2), colored by either cell subsets (top) or the continuous pseudotime (bottom).

E. Dot plots that show the change in the overall cytotoxicity score, exhausted score, the ratio of two scores, respectively, with increasing pseudotime.

F. Box plots that respectively show the cytotoxicity score, exhausted score, the ratio of two scores, in each T cell subset.

G. Heatmap that shows the change in expression level of representative top genes that are related to cell development, with increasing pseudotime. These genes are grouped as five color-coded clusters.

H. Density plot that shows the change in the distribution of cells from different histotypes of tumours, with increasing pseudotime.

I. Density plot that shows the change in the distribution of cells from tumours with different ITH degree, with increasing pseudotime.

J. Transcriptional trajectory of T cells from Monocle (version 2), colored by seven pseudotime stages as indicated in the annotation bar (top). The proportion of T cells with different pseudotime stage in each group of samples with different ITH degree, is shown at the bottom histogram.


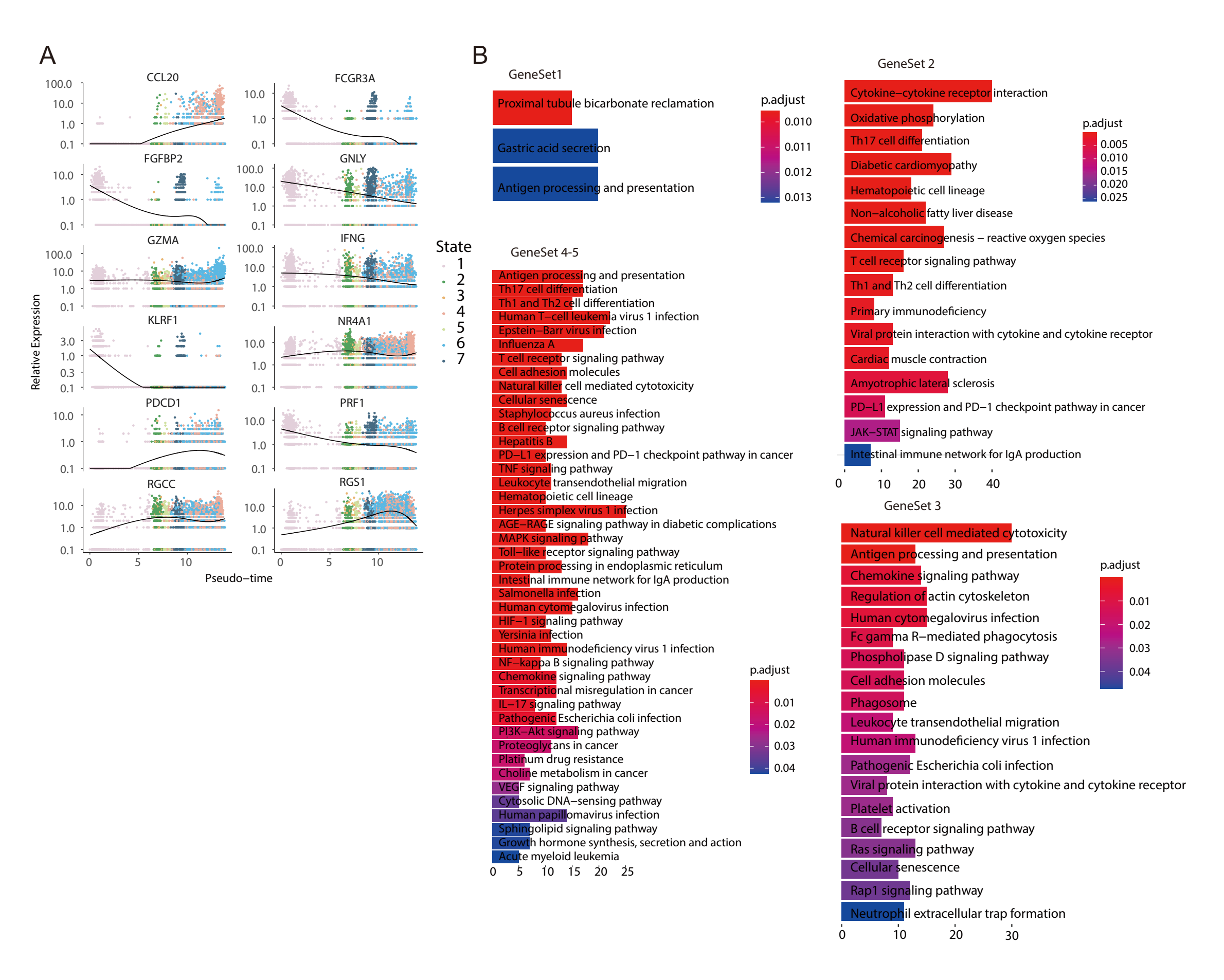


### Figure S7. Function analysis for different pseudotime periods during T cell development.

A. The expression of representative top genes that are related to cell development, with increasing pseudotime. B. Enrichment of different KEGG pathways using gene sets which were associated with T cell development is shown in GeneSet 1, GeneSet 2, GeneSet 3, GeneSet 4-5, respectively.


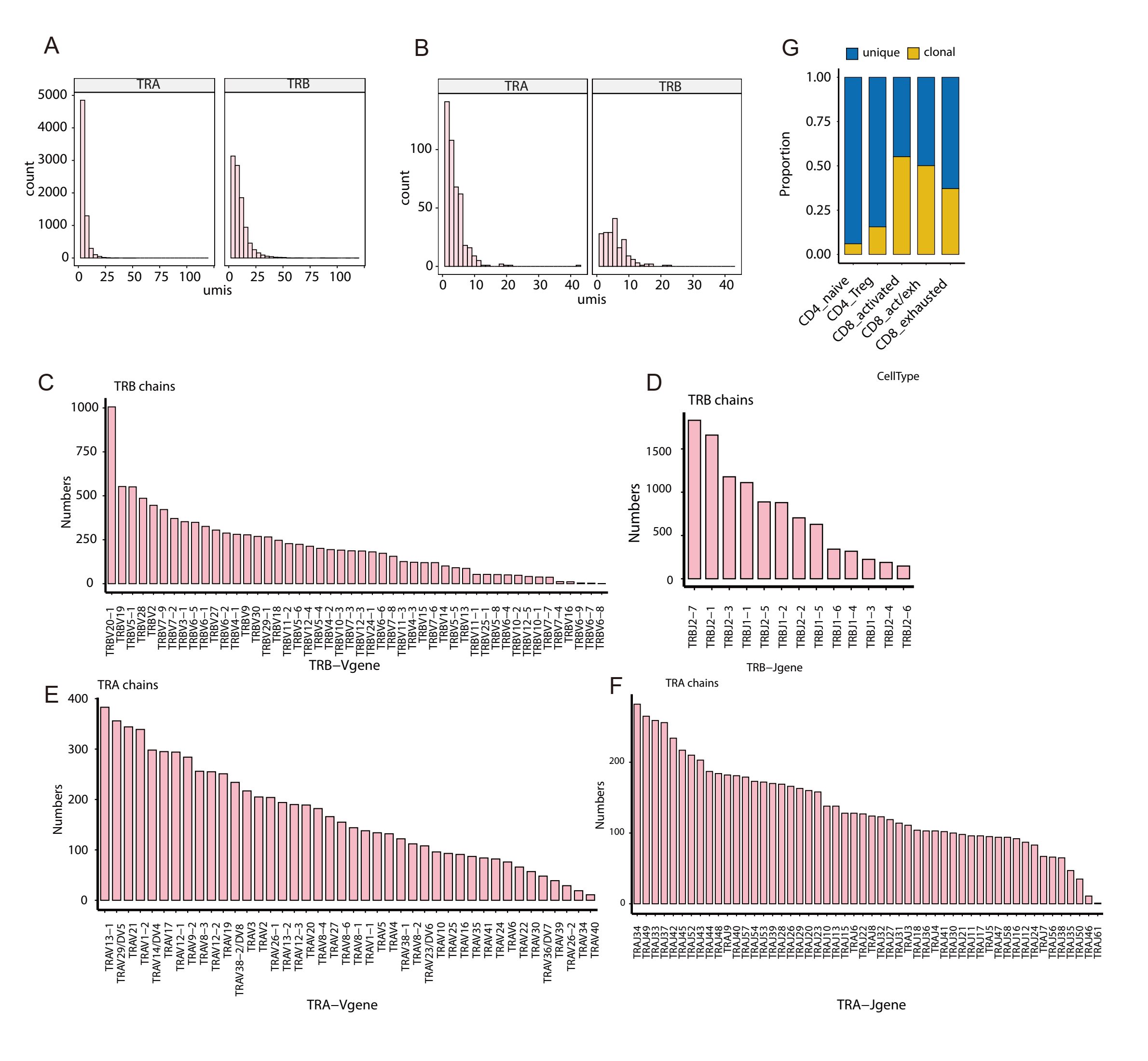


### Figure S8. The profile of TCR usage in GAC-related T cells.

**A.** The distribution of abundance of TCR α- or β- chain expressed in dominant single T cells.

**B.** The distribution of abundance of TCR α- or β- chain expressed in undominant single T cells.

**C-F.** Bar plots show usage of expressed V- (**C**)and J- (**D**) alleles of TCR beta chain in all T cells. Bar plots show usage of expressed V- (**E**) and J- (**F**) alleles of TCR alpha chain in all T cells.

**G.** The proportion of unique or clonal T cell clone in T cell sublinage.


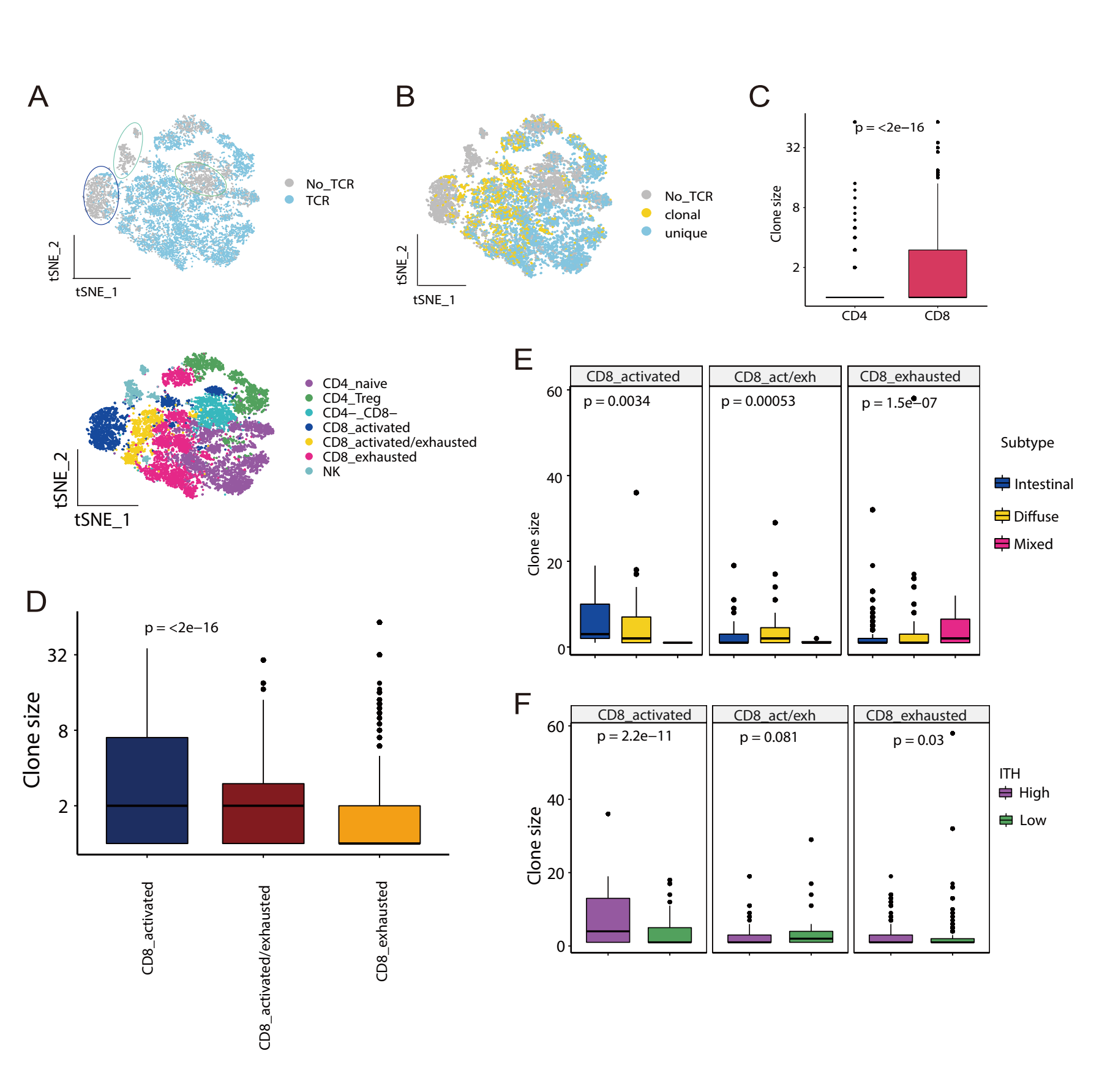


### Figure S9. Clonal dynamics and phenotype transitions of ITH-H tumor-derived T cells.

A. t-SNE plot of 13,358 T cells color-coded by TCR status and seven cell subsets (bottom). Only 9,017 of these T cells had TCR information.

B. T cells with TCR information are further classified as either clonal or unique.

C. Boxplot that shows the difference of clone size between CD4+ T cells and CD8+ T cells.

D. Boxplot that shows the difference of clone size among CD8+ activated, CD8+ activated/exhausted and CD8+ exhausted T cells.

E. Boxplot that shows the difference of clone size across histotypes in CD8+ activated group, CD8+ activated/exhausted group, CD8+ exhausted group, respectively.

F. Boxplot that shows the difference of clone size across different ITH degree in CD8+ activated group, CD8+ activated/exhausted group, CD8+ exhausted group, respectively.


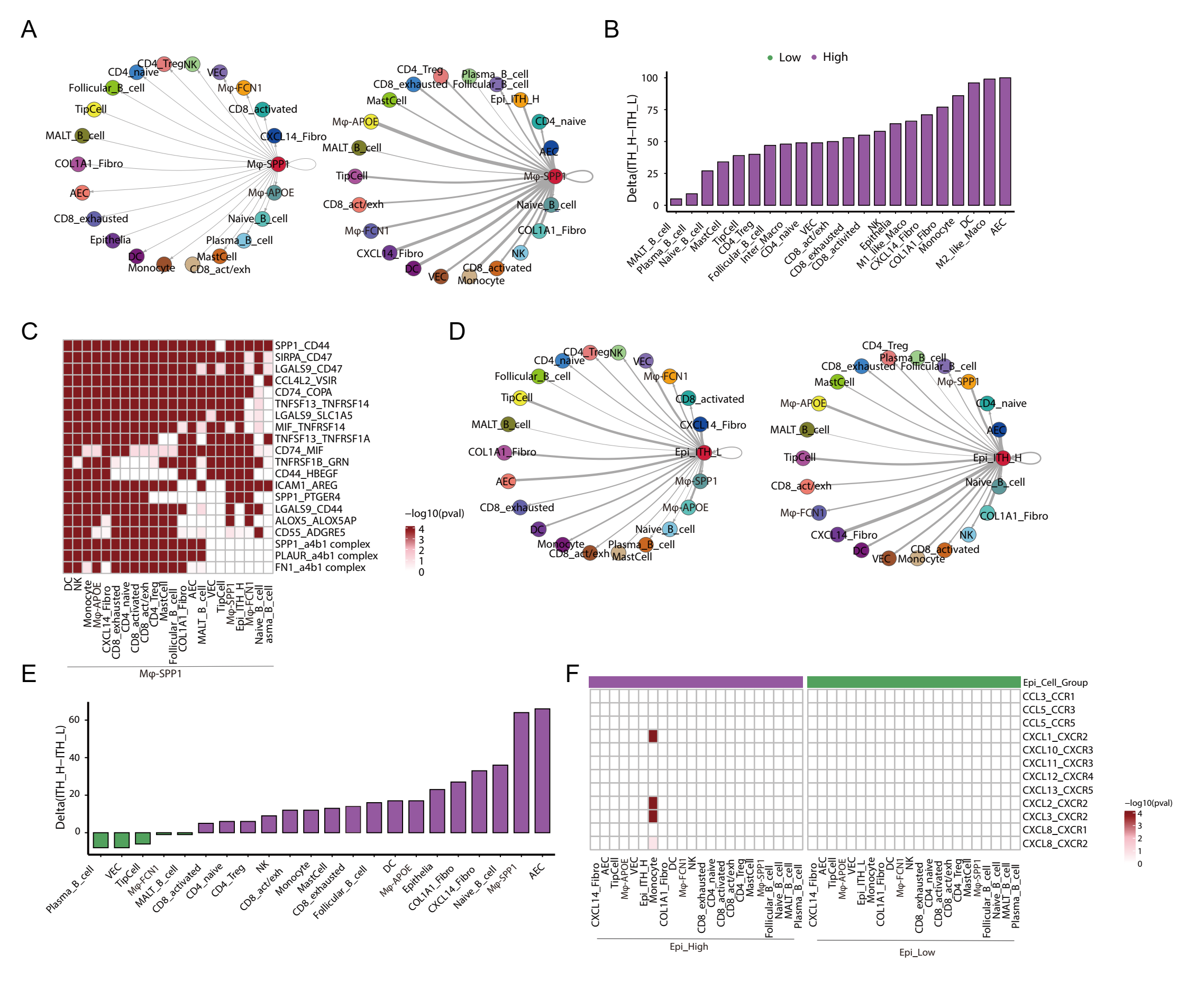


### Figure S10. Intercellular network analysis uncovered the divergent crosstalk among cells in GAC.

A. Predicted intercellular networks centered on Mφ-SPP1 (the intermediate macrophage) macropahage lineage derived from either ITH-L samples (left) or ITH-H samples (right).

B. The intensity gap (y-axis: delta = Intensity _ITH-H_ – Intensity _ITH-L_) of interaction between Mφ-SPP1 macrophage and the other cell lineages (x-axis: cell lineages other than Mφ-SPP1 macrophage). Purple color: The intensity of a particular interaction among a pair of cell lineages is higher in ITH-H group comparing to ITH-L group; the reverse result is represented by green color.

C. The top 20 significant ligand-receptor pair genes (y-axis) accounting for specific interactions between Mφ-SPP1 macrophage and the other cell lineages (x-axis).

D. Predicted intercellular networks centered on the epithelial cancer cell lineage derived from either ITH-L samples (left) or ITH-H samples (right).

E. The intensity gap (y-axis: delta = Intensity _ITH-H_ – Intensity _ITH-L_) of interaction between epithelial cancer cell lineage and the other cell lineages (x-axis: cell lineages other than epithelial cell). Purple color: The intensity of a particular interaction among a pair of cell lineages is higher in ITH-H group comparing to ITH-L group; the reverse result is represented by green color.

F. Heatmap that shows the ligand-receptor pairs of chemokines between epithelial cancer cell lineage and other cell lineages. Result is split into two parts according to the ITH degree classification. Significant ligand-receptor pairs are highlighted by red color.
